# Engineering RNA export for measurement and manipulation of living cells

**DOI:** 10.1016/j.cell.2023.06.013

**Published:** 2023-07-11

**Authors:** Felix Horns, Joe A. Martinez, Chengcheng Fan, Mehernaz Haque, James M. Linton, Victoria Tobin, Leah Santat, Ailiena O. Maggiolo, Pamela J. Bjorkman, Carlos Lois, Michael B. Elowitz

**Affiliations:** 1 Division of Biology and Biological Engineering, California Institute of Technology, Pasadena, CA 91125; 2 Howard Hughes Medical Institute, California Institute of Technology, Pasadena, CA 91125; 3 Division of Chemistry and Chemical Engineering, California Institute of Technology, Pasadena, CA 91125

## Abstract

A system for programmable export of RNA molecules from living cells would enable both non-destructive monitoring of cell dynamics and engineering of cells capable of delivering executable RNA programs to other cells. We developed genetically encoded cellular RNA exporters, inspired by viruses, that efficiently and selectively package and secrete target RNA molecules from mammalian cells within protective nanoparticles. Exporting and sequencing RNA barcodes enabled non-destructive monitoring of cell population dynamics with clonal resolution. Further, by incorporating fusogens into the nanoparticles, we demonstrated delivery, expression, and functional activity of exported mRNA in recipient cells. We term these systems COURIER (Controlled Output and Uptake of RNA for Interrogation, Expression, and Regulation). COURIER enables measurement of cell dynamics and establishes a foundation for hybrid cell and gene therapies based on cell-to-cell delivery of RNA.

## IN BRIEF –

The development of synthetic extracellular vesicles, based on designed protein nanocages, for the programmable and efficient export, cell-to-cell delivery, and expression of mRNA.

## Graphical Abstract

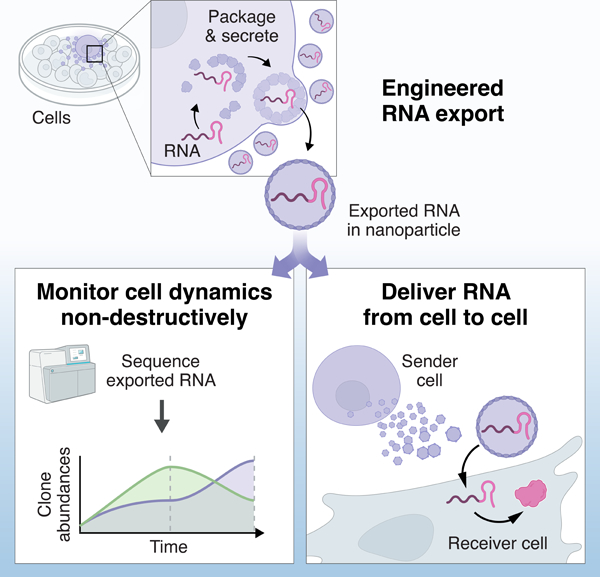

## Introduction

As a central information carrier in the cell, RNA provides a powerful interface for reading and writing cell behaviors. Sequencing RNA enables readout of cell states. In parallel, expression of RNA controls cell states. However, RNA is typically confined within the cell that produced it, limiting its utility for molecular analysis and intercellular communication. By contrast, the ability to programmably export RNA molecules from cells could unlock ways to both analyze and control living cells.

**Figure 1.**
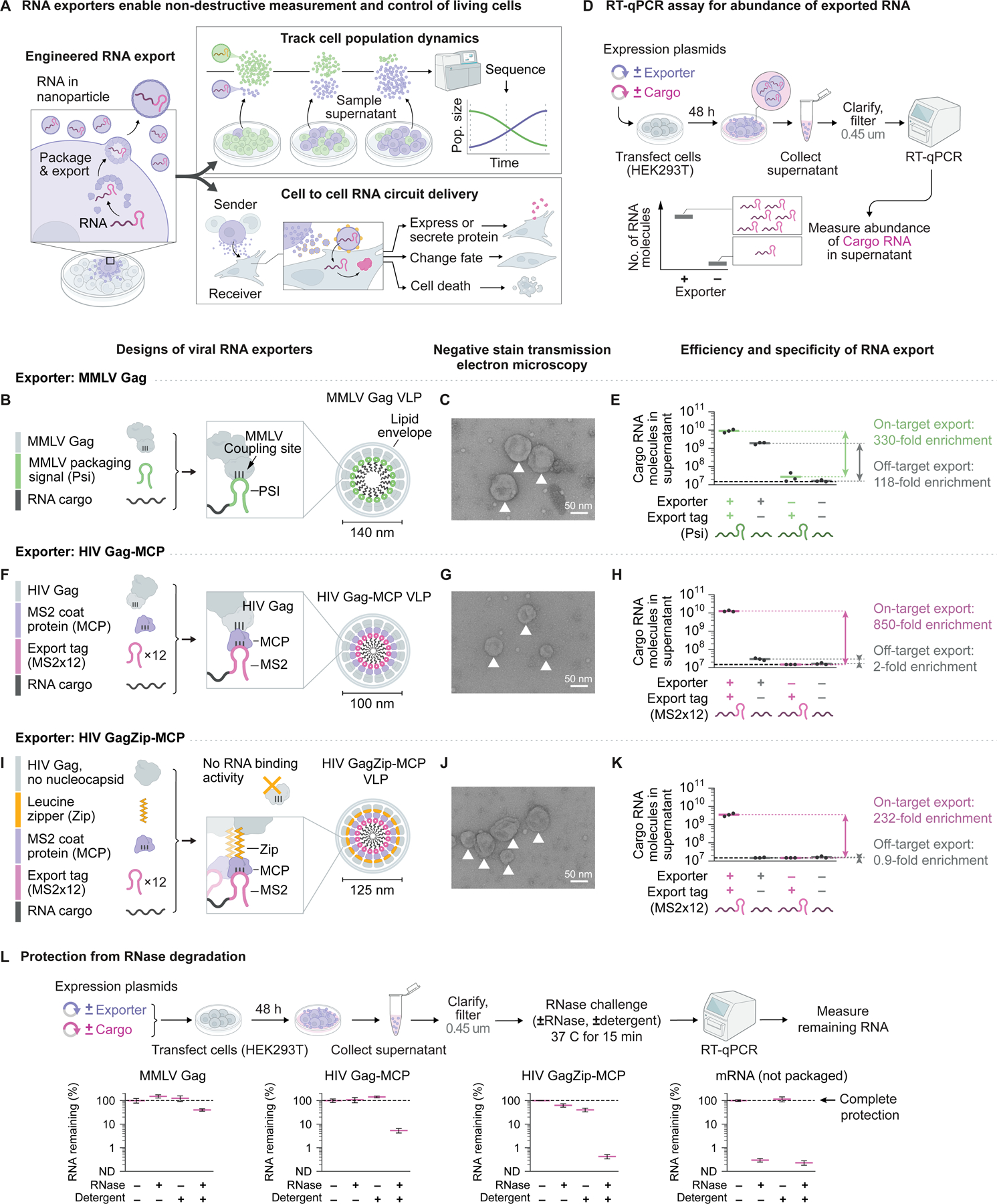
Engineered viral RNA exporters package, secrete, and protect RNA. (A) RNA export enables non-destructive tracking of cell populations and cell-to-cell delivery of RNA. For delivery, exporter nanoparticles incorporate fusogens (yellow dots). (B, F, and I) Designs of viral RNA exporters, in which a capsid fused to an RNA binding domain packages RNA cargo bearing an export tag into virus-like particles (VLPs) that are secreted from cells. (C, G, and J) Cells expressing RNA exporters secreted VLPs (marked by arrowheads), as visualized by electron microscopy of purified culture supernatant. (D) Schematic of assay for abundance of exported RNA. (E, H, and K) Engineered RNA exporters secreted RNA bearing export tags efficiently with varying specificity. Each dot represents one technical replicate; solid line indicates mean of replicates. Black dashed line indicates lower limit of quantification. (L) Top: Schematic of RNase protection assay. Bottom: Exporters protected RNA from degradation by RNases. Unpackaged mRNA was not protected (right). Data for (L) are mean and standard deviation of three technical replicates. See also Figure S1 and Figure S4.

RNA export enables non-destructive measurement of cell dynamics. Single-cell RNA sequencing and hybridization-based assays have revolutionized biomedicine by enabling researchers to decipher the molecular types and states of individual cells at genome scale^1–4^. However, physically accessing RNA for analysis generally requires lysis or fixation of cells, preventing one from tracking the dynamic behavior of individual living cells over time. Cell-free RNA is naturally secreted by cells in extracellular vesicles or upon cell death, and sequencing this RNA can non-destructively reveal biomarkers of health and disease^5–7^. However, the low rates of natural RNA secretion^8,9^ limit the sensitivity and information content of cell-free RNA assays. As an alternative approach, engineering cells to efficiently export RNA molecules that encode information about cell populations and states, then collecting and sequencing this exported RNA could enable non-destructive measurement of cell dynamics with enhanced sensitivity and information content compared to natural cell-free RNA assays (Figure 1A).

RNA export also unlocks ways to manipulate cell behaviors. The ability of RNA to encode proteins and regulate gene expression promises programmable control of cell behaviors. However, therapeutic use of this capability remains limited by challenges in delivering RNA to specific cell populations within tissues^10^. The ability to engineer cells to export RNA raises the possibility of creating therapeutic “delivery cells” that home to tissues, recognize target cells, and locally deliver RNA circuits that execute diverse functions within recipient cells, including altering their gene expression, reprogramming cell fate, or selectively killing cells in diseased states^11^ (Figure 1A). This strategy could circumvent difficulties encountered with other delivery vectors in achieving tissue and target specificity, as cells are capable of penetrating tissues and utilizing cell-based sensing and logic to conditionally regulate localized RNA delivery. A foundational component of this vision is a system that efficiently exports RNA cargo within a vehicle that permits uptake and expression of the RNA by non-engineered receiver cells.

Virus-like particles (VLPs) and extracellular vesicles (EVs) are attractive platforms for export and delivery of RNA. Viral structural proteins (also known as capsids) and their natural interactions with RNA packaging signals have been used to package and transfer RNA between cells in VLPs^12–15^. However, these approaches have often relied on retroviral capsid proteins, such as those of human immunodeficiency virus (HIV) or Moloney murine leukemia virus (MMLV), which exhibit only modest binding specificity for viral RNA, as they readily bind other RNA^16–20^, posing challenges for specific loading of cargo RNA. VLPs^21,22^ and EVs^23–25^ have been engineered to improve the selectivity of cargo RNA loading, including by fusing RNA binding proteins to capsids or proteins incorporated into EVs, and tagging cargo RNA with cognate interacting sequences. These approaches have allowed RNA delivery, including *in vivo* in mice^21,24–26^, but require further development, as they have been limited by inefficient cargo loading and secretion^23–25^; restricted cargo capacity and poor cargo expression after delivery^23^; or capsid modifications that impair VLP assembly^27,28^ and likely hinder secretion^21^.

An ideal RNA export system would overcome these limitations and provide several key features. First, it would export RNA from mammalian cells efficiently, thereby allowing sensitive measurement and potent delivery. Second, it would permit selective export of target RNAs, such as engineered barcodes or cargos. Third, it would protect the exported RNA from degradation by extracellular RNases. Fourth, it would enable delivery and expression of cargo RNA in recipient cells. Finally, expression of export system components would be minimally perturbing to the expressing cells. An RNA export system having these features could be used to create versatile RNA-based reporter and delivery platforms.

Here, we report the development of RNA export systems having these features, and the application of these systems for non-destructive monitoring of cell population dynamics and cell-to-cell delivery of mRNA. We designed a set of RNA exporters that combine three types of modular protein components: (i) RNA binding proteins to capture specific RNA molecules, (ii) self-assembling capsids or vesicles to package and secrete those RNAs, and (iii) fusogens to deliver secreted RNA to target cells. We engineered several generations of RNA exporters, starting from VLPs and culminating in extracellular vesicles based on protein nanocages, which efficiently packaged and secreted RNA from cells and exhibited progressive improvements in selectivity for target RNA. We then combined RNA export with genetic barcoding and sequencing to non-destructively monitor cell population dynamics. Finally, by incorporating fusogens into the secreted nanoparticles, we demonstrated delivery and functional activity of cargo RNA in target cells, including mRNA encoding Cre recombinase and fluorescent proteins. We term these systems COURIER, for Controlled Output and Uptake of RNA for Interrogation, Expression, and Regulation. These results establish COURIER as a flexible and extensible paradigm for non-destructive measurement of cell dynamics and intercellular transfer of RNA.

## Results

### Engineered viral RNA exporters package, secrete, and protect RNA

Because RNA viruses are naturally efficient RNA exporters, we initially sought to engineer RNA exporters based on viral components. We focused on retroviral capsid proteins, which self-assemble to form secreted VLPs when expressed in mammalian cells^29^. Retroviruses package their genomes into viral particles via interactions between capsid proteins and RNA structures that serve as packaging signals^30^.

We first verified that repurposed retroviral components enable export of RNA from mammalian cells, as shown in classic work^30^. We transiently expressed the capsid protein (Gag) from MMLV with cargo RNA that was tagged in its 3’ untranslated region (3’ UTR) with the MMLV packaging signal (PS) in human embryonic kidney (HEK293T) cells (Figure 1B). These cells secreted spherical VLPs of ~140 nm diameter, as characterized by negative-stain transmission electron microscopy (Figure 1C) and dynamic light scattering (Figure S1A). Similar particles were absent in the supernatant of cells expressing only target RNA, but not exporter (Figure S1B).

To quantify RNA export, we measured the abundance of cargo RNA in cell culture supernatant using reverse transcription and quantitative polymerase chain reaction (RT-qPCR). We collected supernatant, clarified it by centrifugation and filtered it to remove cells and large debris, extracted RNA, treated with DNase, and finally performed RT-qPCR for the target RNA (Figure 1D). The cleanup steps of clarification and filtration reduced RNA recovery by 36% and 55% respectively, leading to a combined loss of 64% (Figure S1C), but enabled stringent measurement of bona fide RNA export. This assay faithfully and reproducibly measured the abundance of RNA in supernatant, rather than potential DNA contaminants such as expression plasmids (Figure S1D). The MMLV Gag system efficiently exported cargo RNA. MMLV PS-tagged cargo RNA was enriched 330-fold in supernatant in the presence compared to the absence of Gag (Figure 1E). However, we also observed PS-independent export: non-target RNA, lacking the PS tag, was enriched 118-fold in supernatant in the presence of Gag. This non-specific export activity can be explained by the broad RNA binding activity of the MMLV Gag nucleocapsid domain^18,19^, a property shared by other retroviral capsid proteins^16,17,19,20^. Thus, retroviral components export RNA efficiently, but possess only partial specificity for tagged cargo RNA.

To improve the targeting specificity of RNA export, we replaced the native RNA recognition system with a more specific alternative. We focused on the well-characterized sequence-specific RNA binding protein, MS2 bacteriophage coat protein (MCP), which binds to a cognate RNA hairpin aptamer^31^. To facilitate engineering, we replaced the MMLV Gag capsid with the HIV Gag capsid, which has better annotated functional domains^32^ and tolerates protein fusions without compromising VLP assembly and release^22,33^. We fused MCP to HIV Gag, forming Gag-MCP, and tagged cargo RNA with twelve tandem MS2 hairpins in its 3’ UTR (Figure 1F). Although export systems based on fusions of HIV Gag and MCP were previously developed^21,22^, they used protein architectures that inhibit VLP assembly^27,28^, and their export efficiency and targeting specificity were not quantitatively characterized.

HEK293T cells transiently expressing these components secreted spherical VLPs of ~100 nm diameter (Figure 1G and Figure S1A), similar to immature HIV particles^34^. This Gag-MCP system efficiently exported MS2-tagged cargo RNA, which was enriched 850-fold in supernatant in the presence compared to the absence of Gag-MCP (Figure 1H), as assayed by RT-qPCR, similar to the MMLV Gag system. However, the engineered Gag-MCP system exhibited greater specificity than MMLV Gag: non-target RNA, lacking the MS2 tag, was enriched only 2-fold in the presence of Gag-MCP. Altogether, the Gag-MCP system achieved similar export efficiency and substantially greater export specificity compared to MMLV Gag.

To quantify export rates, we monitored the accumulation of RNA in supernatant over time after transfection. RNA accumulated at an average rate of 1,012 ± 52 (mean ± s.d.) molecules per cell per hour (Figure S1E). This rate could be tuned by altering the number of tandem repeats of the MS2 tag in the target RNA (Figure S1F). Because efficiency saturated at the maximum rate with 8 or more repeats, we used 8 repeats for most subsequent experiments. Export rates also varied with expression levels of the exporter (Figure S1G) and target RNA (Figure S1H). Finally, the system functioned when stably expressed from genomically integrated transgenes, achieving 40-fold enrichment of cargo RNA in culture supernatant (Figure S1I and Methods). This enrichment was less than the 850-fold enrichment achieved by transient expression (Figure 1H), likely due to lower levels of exporter and cargo RNA expression from integrated transgenes. Taken together, these results show that fusing sequence-specific RNA binding proteins to retroviral capsids enables efficient, specific, and tunable export of target RNA.

Despite the improved specificity of Gag-MCP, it remained possible that non-specific export could be suppressed even further. The nucleocapsid domain of HIV Gag (like that of MMLV Gag) binds diverse RNA sequences^19,20^. We reasoned that ablating its RNA binding activity could reduce non-specific RNA packaging, while preserving the sequence-specific targeting conferred by MCP. However, the RNA binding activity of Gag is essential for nucleating viral particle assembly^27,28^. Indeed, deleting the critical zinc finger RNA binding motif (ZF2) from Gag-MCP, forming GagΔZF2-MCP, strongly reduced export activity to background levels (Figure S1J and Figure S1K), consistent with failure of particle assembly. These results suggest a limitation on capsid engineering arising from the lack of modularity of RNA binding and self-assembly, and provoke the question of whether protein engineering could separate these functions.

This defect in viral assembly was previously shown to be rescued by addition of a leucine zipper homooligomerization domain^35^. To test whether a similar design could rescue GagΔZF2-MCP particle assembly and enable specific RNA export, we substituted a leucine zipper for the nucleocapsid domain in Gag-MCP, to form a new construct, denoted GagZip-MCP (Figure 1I). Cells expressing GagZip-MCP secreted ~125 nm particles (Figure 1J and Figure S1A) and efficiently exported MS2-tagged cargo RNA with undetectable export of non-target RNA lacking the MS2 export tag (Figure 1K). We screened several other designs (Figure S1J), including an exceptionally compact design that we termed MiniGagZip-MCP, in which domains previously found to be non-essential for particle assembly and release were deleted^36^. However, most of these designs did not robustly export RNA (Figure S1K). We confirmed that differences in export efficiency were not due to differences in expression of cargo RNA (Figure S1L). Thus, through iterative engineering of viral RNA exporters, we reduced non-specific export activity to undetectable levels, while maintaining high export efficiency.

To be utilized, exported RNA must be protected from degradation by RNases. To measure protection from RNase activity, we exported cargo RNA, collected and filtered culture supernatant, challenged it with a mixture of RNases A and T1, and quantified the remaining RNA using RT-qPCR (Figure 1L). RNA secreted by all three viral exporters was completely protected from degradation during RNase challenge (Figure 1L). By contrast, *in vitro* transcribed mRNA, which was not packaged in VLPs, was degraded substantially. When we added detergent to disrupt lipid envelopes, protection from RNase was substantially reduced, confirming that RNA was packaged within lipid-enveloped VLPs (Figure 1L). Interestingly, unlike the other exporters, MMLV Gag provided some RNA protection even in the presence of detergent, possibly reflecting its intrinsic RNA binding activity.

Exported RNA was also protected from degradation in culture supernatant and blood. 95% of the RNA packaged and secreted by Gag-MCP remained intact after incubation in culture supernatant at 37 C for 6 days, corresponding to a half-life of 19.0 ± 10.7 days (mean ± s.d.) (Figure S1M and Methods). No degradation of RNA exported by Gag-MCP was detected after 24 hours of incubation in whole mouse blood, which contained cells, at 37 C (Figure S1N). This indicates that any potential loss of RNA due to cellular uptake of particles requires >1 d. Taken together, these results indicate that engineered viral RNA exporters efficiently package, secrete, and protect RNA within enveloped VLPs.

### Engineered protein nanocages package, secrete, and protect RNA

Despite functioning well, the viral exporter designs described so far had limited potential for further engineering. Rational design of viral proteins is difficult because their architectures are not modular^32^, as highlighted by the dual role of nucleocapsid domains in RNA binding and particle assembly in diverse retroviruses^27,28,37,38^. In addition, fusing proteins to viral capsids can disrupt VLP assembly in an unpredictable manner^39^. Finally, viral proteins interact extensively with host proteins^40,41^, including stimulating innate antiviral sensing pathways^42–46^. To overcome these limitations, we sought to engineer synthetic RNA exporters based on designed proteins, which are inherently modular, should tolerate fusion to other proteins^47^, and are not expected to stimulate antiviral sensors^42–46^.

**Figure 2.**
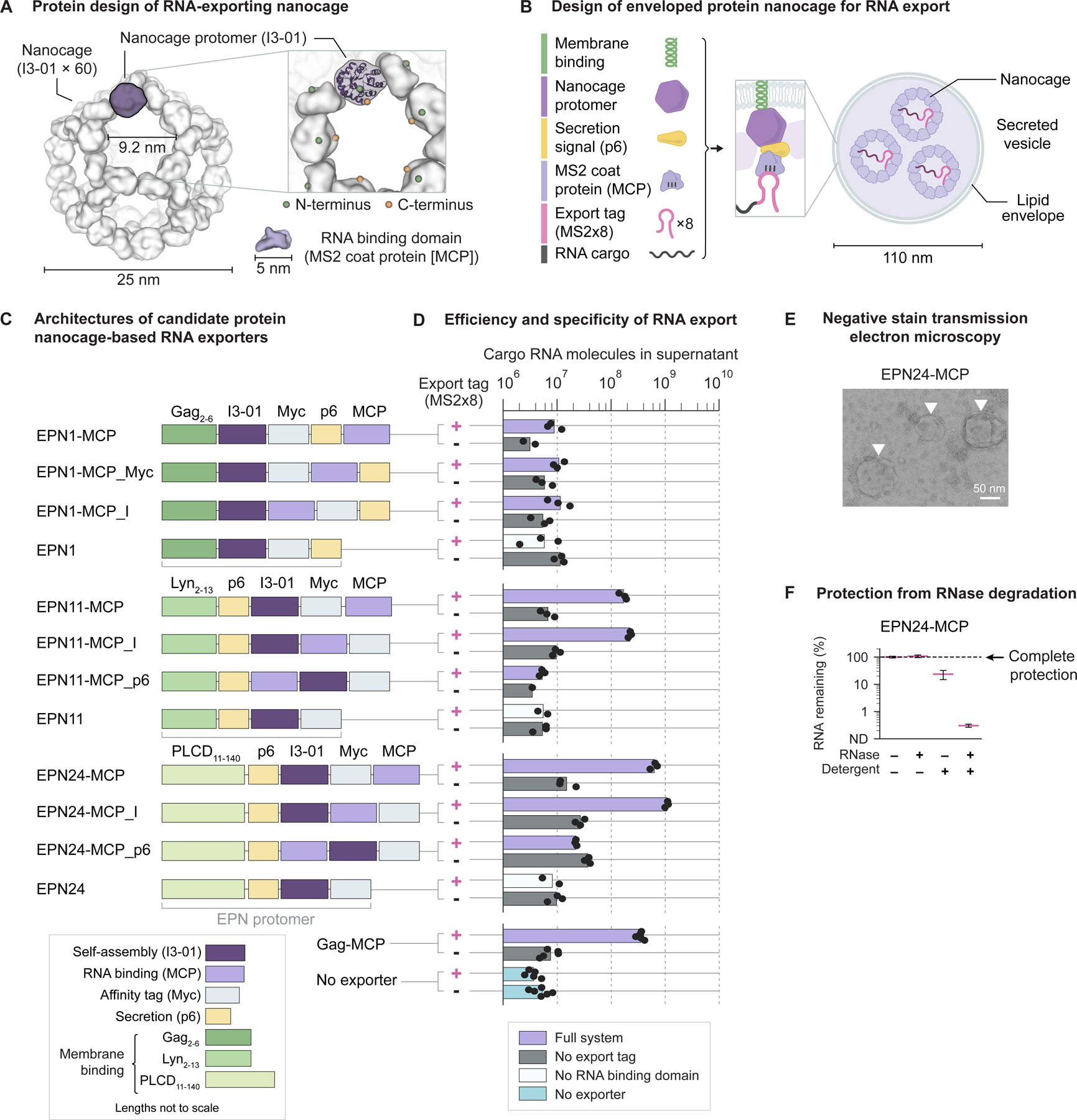
Engineered protein nanocages package, secrete, and protect RNA. (A) Design model of nanocage^48^ (PDB 5KP9) has cavities that accommodate the RNA binding protein MCP^77^ (PDB 1MSC). N- and C-termini of nanocage protomer (marked by spheres) are surface-exposed and oriented towards the cavity. (B) Design of nanocage-based RNA exporter, in which self-assembling protomers fused to an RNA binding domain package RNA cargo bearing the MS2 export tag (8 MS2 hairpins, denoted MS2×8) into vesicles that are secreted from cells (schematic). (C) Architectures of candidate nanocage-based exporters. (D) Cells expressing nanocage-based exporters efficiently and specifically secreted RNA bearing export tags into culture supernatant, as measured by RT-qPCR. Each dot represents one technical replicate and bar indicates their mean. (E) Cells expressing EPN24-MCP secreted vesicles (marked by arrowheads), as visualized by electron microscopy of purified culture supernatant. (F) Nanocage-based exporters protected RNA from degradation by RNase. Data for (F) are mean and standard deviation of three technical replicates. See also Figure S2 and Figure S4.

Enveloped protein nanocages (EPNs) represent a spectacular achievement of protein design and provide an ideal foundation for RNA export. EPNs are composed of designed protomers that self-assemble into 60-subunit dodecahedral “nanocages”^47^. Addition of a membrane binding domain and a secretion signal (the p6 peptide from HIV) enables their secretion from mammalian cells within extracellular vesicles, which average ~110 nm in diameter and each contain ~20 nanocage assemblies^48^. Further, EPN protomers with diverse domain components and orderings have been secreted from cells^48^. While the ability of EPNs to package and export RNA has not been reported, their design includes cavities large enough to accommodate RNA binding proteins (Figure 2A). Both the N- and C-termini of the nanocage protomer are surface-exposed and oriented towards a cavity, suggesting that additional domain fusions may be tolerated.

To test whether EPNs can export RNA, we designed 9 nanocage variants, based on 3 distinct EPN protomer architectures, which possess different membrane binding domains, with MCP fused at 3 different positions within each architecture (Figure 2B and Figure 2C). When expressed together with MS2-tagged cargo RNA, four variants efficiently exported RNA, comparable to the most efficient viral exporter Gag-MCP (Figure 2D). Export was specific for tagged cargo RNA, as non-target RNA lacking the export tag was secreted at substantially lower rates. Variants lacking MCP also did not export target RNA above background rates, indicating that the designed RNA binding interaction is required for export.

Among these variants, EPN24-MCP exhibited the highest export efficiency. The rate of export by EPN24-MCP was similar to that of Gag-MCP, as indicated by accumulation of RNA over time after transfection (Figure S2A). As with Gag-MCP, this rate could be tuned by altering the copy number of the MS2 tag in the cargo RNA, and saturated at a maximum rate with 8 or more copies (Figure S2B).

EPN24-MCP packaged RNA in extracellular vesicles. Cells expressing EPN24-MCP secreted vesicles with ~120 nm diameter, as revealed by electron microscopy (Figure 2E and Methods) and dynamic light scattering (Figure S2C). Exported RNA was protected from degradation during RNase challenge (Figure 2F). Protection was abolished by detergent treatment, indicating that RNA was packaged within lipid-enveloped particles. The relative number of RNA molecules encapsulated per particle increased with the number of MS2 tags, saturating at ~8 tags (Figure S2B, Figure S2D, and Methods). The cargo capacity of the EPN24-MCP system reaches at least 9.8 kb (Figure S2G, Figure S2H, Figure S2I, and Methods).

RNA encapsulated by EPN24-MCP was protected from degradation in cell culture supernatant and blood. 36% of the RNA exported by EPN24-MCP remained intact after incubation in culture supernatant at 37 C for 6 days (Figure S2E). No degradation of RNA exported by EPN24-MCP was detected after 24 hours of incubation in whole mouse blood at 37 C (Figure S2F).

EPNs were designed for secretion via the ESCRT (endosomal sorting complex required for transport) pathway^48^. To test whether export rates are ESCRT-dependent and whether they could be further enhanced, we selected a panel of six modulators of virus or exosome secretion^49–51^, of which two, NEDD4L and CIT, were previously shown to enhance HIV budding by promoting recruitment of ESCRT-I to HIV p6 domains^49,50^. We co-expressed each candidate modulator together with EPN24-MCP and target RNA, and assayed export by RT-qPCR (Figure S2J). NEDD4L and CIT each enhanced RNA export rates by up to 387% (Figure S2K). This enhancement required both the exporter and the export tag, indicating that these modulators act on EPN24-MCP secretion pathways. By contrast, the other four modulators, which did not enhance export, are not known to affect ESCRT-dependent secretion. Conversely, co-expression of a dominant negative inhibitor of the ESCRT pathway^52^, VPS4-E228Q, abolished export (Figure S2L). These results indicate that EPN24-MCP is secreted via the ESCRT pathway, as designed, and suggest that ESCRT-mediated budding is a rate-limiting step for RNA export.

Finally, we asked whether the RNA targeting domain could be swapped by replacing MCP with an alternative sequence-specific RNA binding domain, the PP7 bacteriophage coat protein. The PP7-based design exported PP7-tagged cargo RNA, demonstrating modular engineering of RNA targeting specificity (Figure S2M and Methods).

Taken together, these results demonstrate that engineered nanocages enable efficient, specific, and programmable export of RNA.

### Genome-scale characterization of RNA export specificity and bias

**Figure 3.**
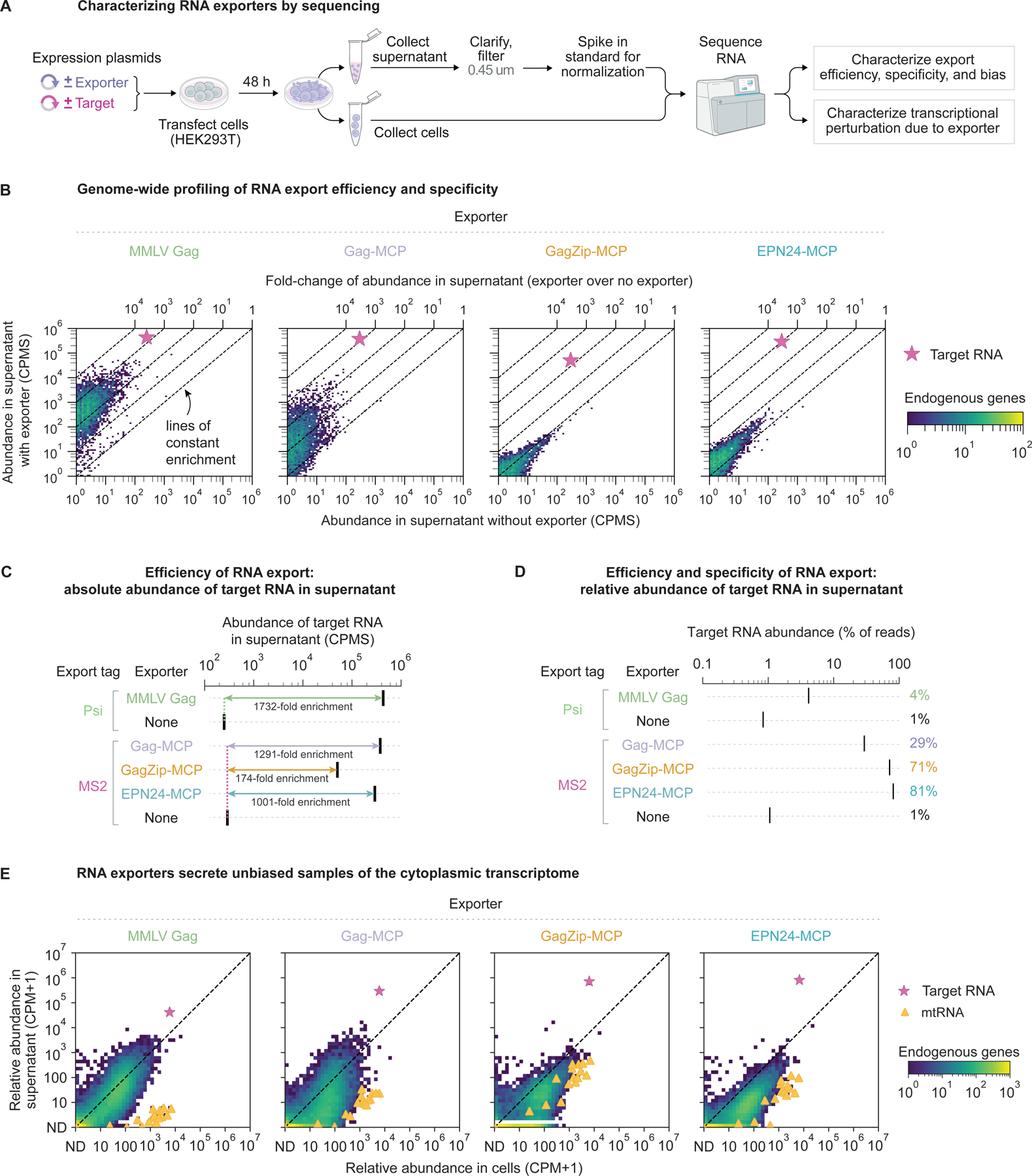
Genome-scale characterization of RNA export. (A) Workflow for sequencing-based analysis of RNA export efficiency, specificity, and bias. (B) Abundance of target RNA and non-target (endogenous) RNA in culture supernatant with and without expression of exporters. Iterative engineering (left to right) of RNA binding properties of exporters reduced non-target RNA enrichment, reflecting improved specificity, while efficient export of target RNA bearing export tags (star) was maintained. (C) Target RNA was enriched in culture supernatant in the presence of each exporter (compared to without the exporter). In (B) and (C), CPMS denotes counts per million of spike-in standard. (D) Engineering improved the specificity of exporters, thereby increasing the fractional abundance of target RNA among total supernatant RNA. (E) Transcript abundances were strongly correlated in the cellular and supernatant transcriptomes, indicating that exporters secreted unbiased samples of the cellular transcriptome. One notable exception was mtRNA, which was depleted in secreted RNA, likely due to its compartmentalization within the mitochondrial lumen. CPM, counts per million (not normalized to spike-in standard); ND, not detected. See also Figure S3.

We next characterized the composition of exported RNA at genome scale using RNA sequencing. We expressed RNA exporters and target RNA by transfecting cells with expression plasmids, clarified and filtered culture supernatant, and sequenced total RNA (Figure 3A). Using spike-in standards to normalize total RNA abundance across samples, we found that the absolute abundance of target RNA bearing export tags was ~1,000-fold higher in supernatant in the presence of exporters MMLV Gag, Gag-MCP, and EPN24-MCP and 174-fold higher in the presence of GagZip-MCP (Figure 3B and Figure 3C), confirming the efficiency of RNA export as measured by RT-qPCR (Figure 1 and Figure 2).

Export specificity improved with each generation of exporter engineering. Target RNA represented only 4% of supernatant RNA reads with the MMLV Gag exporter, but 81% of supernatant RNA reads with the EPN24-MCP exporter (Figure 3D). Improved specificity also manifested as reduced export of endogenous cellular (non-target) RNA, which in aggregate exhibited 345-fold enrichment in supernatant with MMLV Gag (compared to without exporter), but only 2-fold enrichment with EPN24-MCP and negligible enrichment with GagZip-MCP (Figure S3A). These effects were broadly distributed across the transcriptome, as seen by shifts in the full distributions of transcript abundances in supernatant, rather than changes in a subset of transcripts (Figure 3B and Figure S3B). Overall, both GagZip-MCP and EPN24-MCP achieved high specificity (Figure 3D and Figure S3A), exceeding previous estimates based on RT-qPCR analysis of selected transcripts (Figure 1 and Figure 2).

To determine whether non-specific export was biased to favor certain transcripts over others, we compared the composition of exported RNA to that of the cellular transcriptome. Despite varying total amounts of exported endogenous RNA, the relative abundances of genes were strongly correlated between the exported and cellular transcriptomes for all exporters (Figure 3E), indicating that the exporters were largely unbiased among transcripts. One notable exception was mitochondrial RNA (mtRNA), which was strongly depleted in exported RNA, likely due to its compartmentalization within mitochondria^54^. By contrast, few genes were enriched in exported RNA compared to the cellular transcriptome. Gene ontology (GO) analysis of these enriched genes showed no consistent and significant pathway enrichment (Methods). Taken together, these results suggest that, besides cargo and mtRNA, exporters bind, package, and secrete samples of the cytoplasmic transcriptome in an unbiased manner.

The non-specific RNA export activity enhanced the detection rates of endogenous transcripts, including transcripts expressed at low levels and markers of cell identity (Figure S3C and Methods), indicating that engineered export enables non-destructive, cell population level monitoring of transcriptomes. Finally, expression of cargo RNA reduced export of non-cargo RNA, indicating that target and non-target RNA compete for packaging in MMLV Gag and Gag-MCP VLPs (Figure S3D, Figure S3E, and Methods).

### RNA exporters do not perturb cell morphology, growth, or gene expression

For monitoring cell dynamics and transmitting RNA to other cells, the export system should minimally perturb the cell in which it is expressed. Cells expressing exporters from plasmids at levels sufficient to support robust RNA export (Figure 1 and Figure 2) appeared morphologically normal (Figure S4A), exhibited unimpaired growth rates (Figure S4B), and had similar levels of cell death (Figure S4C), compared with cells expressing fluorescent proteins at similar levels. To determine whether RNA exporters perturb endogenous gene expression, we compared cellular transcriptomes after transfecting expression plasmids for fluorescent proteins, either with or without exporters, and observed no significant deviations (Figure S4D), indicating that exporter expression does not perturb endogenous expression differently than a fluorescent protein. Notably, mtRNA transcripts did not change in abundance, suggesting that cell health was maintained^56^. Cells stably expressing the exporter Gag-MCP and cargo RNA from genomically integrated transgenes also appeared morphologically normal (Figure S4E) and did not have elevated rates of cell death (Figure S4F). We conclude that RNA exporter expression is non-toxic and does not detectably perturb cell morphology, growth, or transcriptome state.

### RNA exporters are portable across cell types and species

**Figure 4.**
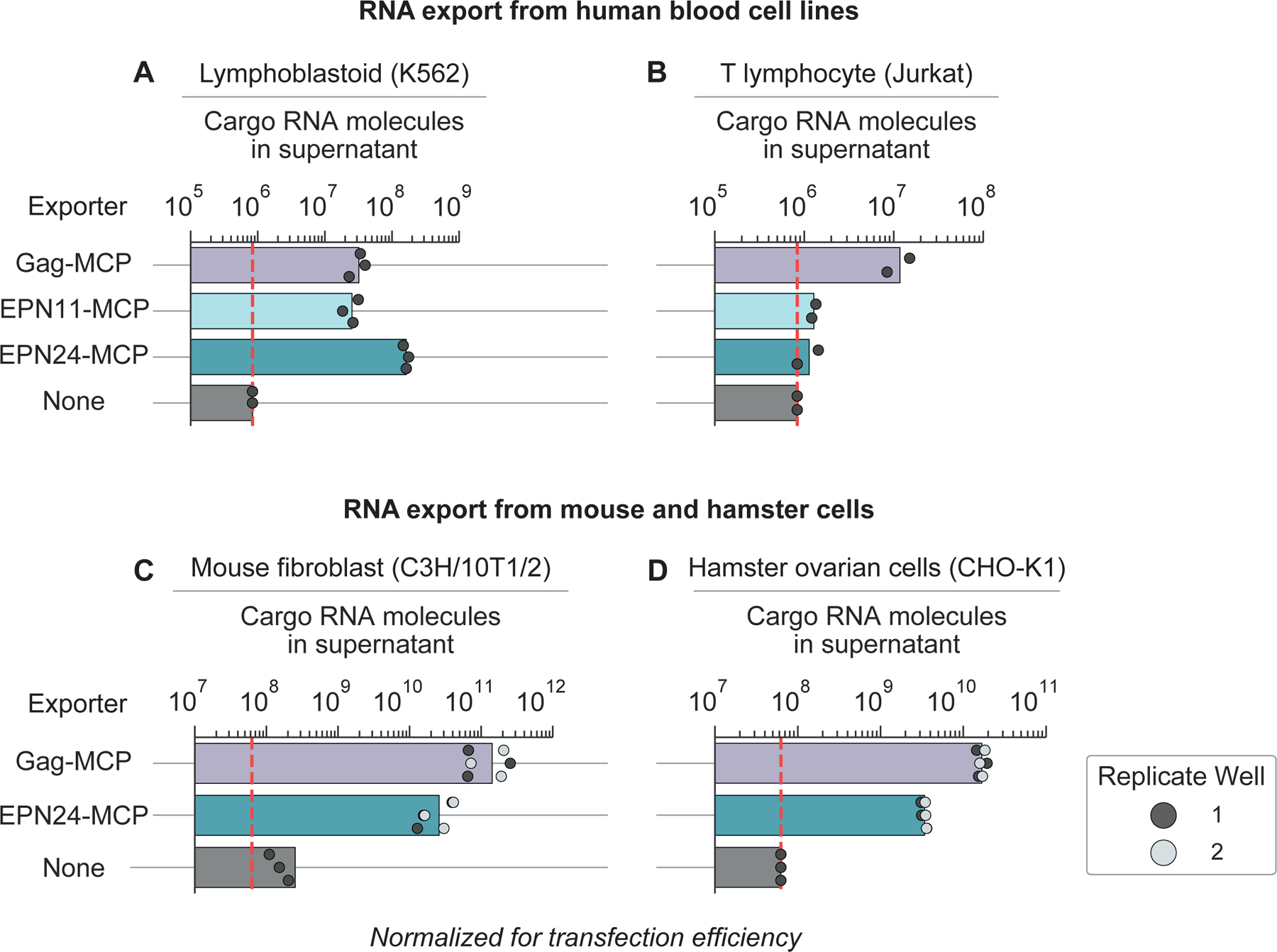
RNA exporters are portable across cell types and species. Human lymphoblastoid (A), human T lymphocyte (B), mouse fibroblast (C), and Chinese hamster ovary (D) cell lines efficiently exported cargo RNA bearing export tags, as measured by RT-qPCR. Each dot represents one technical replicate; colors represent biological replicates (distinct culture wells); bar indicates mean of replicates. Dashed line indicates lower limit of quantification. In (C) and (D), RNA abundances were normalized to account for varying transfection efficiency across cell lines.

The ability to export RNA from human blood cells would unlock applications in cell monitoring and therapeutic delivery. We therefore tested RNA export in lymphoblast cells (K562) and T cells (Jurkat). Gag-MCP, EPN11-MCP, and EPN24-MCP each exported cargo RNA efficiently from K562 cells, with EPN24-MCP achieving the highest efficiency (Figure 4A). By contrast, only Gag-MCP exported RNA efficiently from Jurkat cells (Figure 4B). These results show that engineered RNA exporters can operate in blood cell lines, and suggest that different exporters possess different cell type preferences.

In addition, the ability to export RNA from non-human cells would enable reporting and delivery applications in animal models. Therefore, we tested RNA export in mouse fibroblasts (C3H/10T1/2) and hamster ovary cells (CHO-K1). Both cell lines efficiently exported RNA with export rates similar to those of HEK293 after accounting for transfection efficiency (Figure 4C and Figure 4D). Together, these results demonstrate the portability of RNA exporters across mammalian species and cell types.

### RNA export enables accurate, reproducible, and sensitive monitoring of cell population dynamics

**Figure 5.**
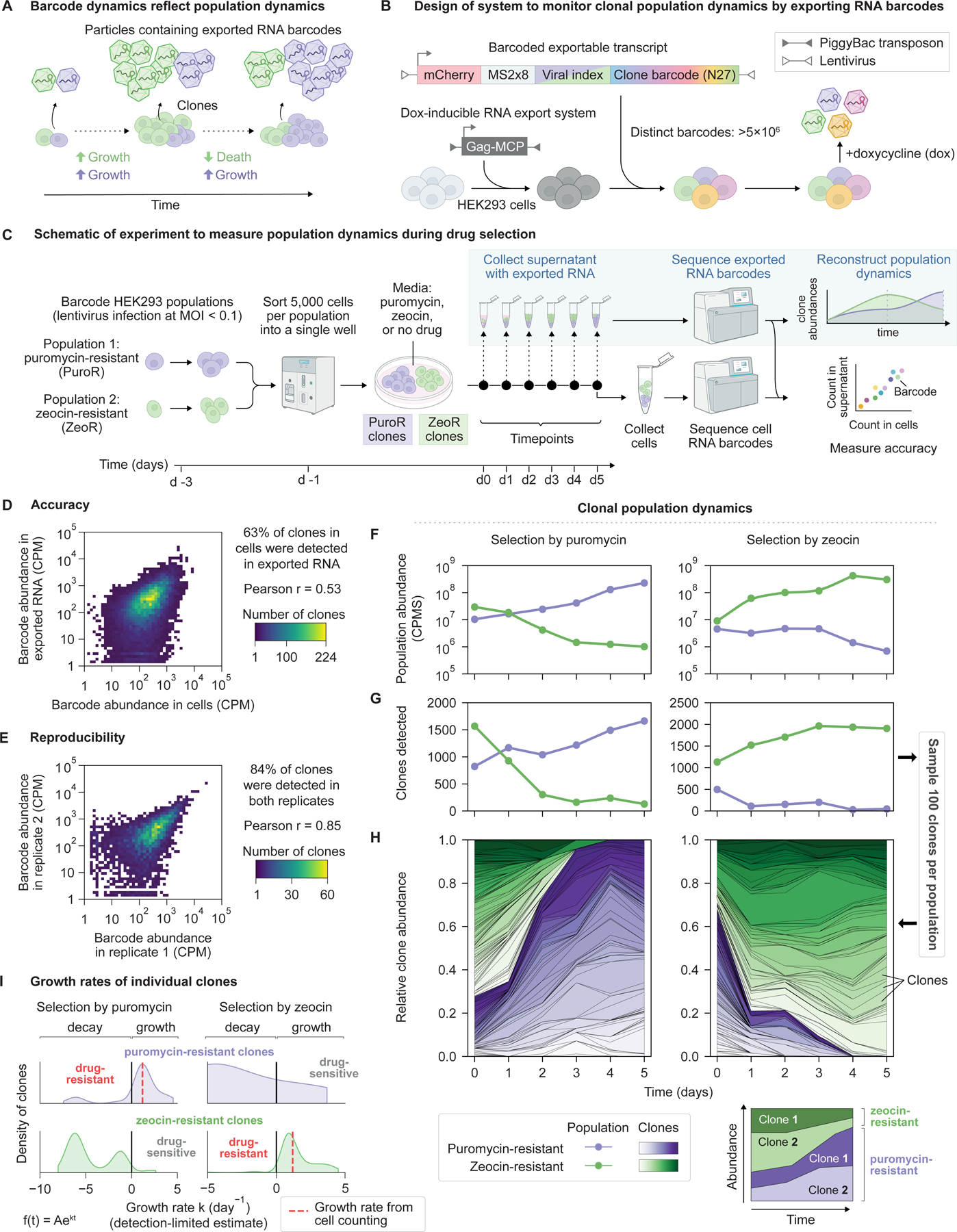
RNA export enables monitoring of mammalian cell population dynamics. (A) Cell population dynamics, including growth and death, can be monitored by longitudinal sampling of exported clonal barcode RNA. (B) Cells were engineered to inducibly express an RNA exporter (Gag-MCP), and genetically barcoded (rainbow) by transduction with a diverse lentiviral library at low multiplicity of infection (MOI < 0.1). Barcode transcripts contained the MS2 export tag. (C) Cells were barcoded, sorted, and cultured for 6 days in the presence of growth-altering drugs. Exported barcodes were collected from supernatant, sequenced, and used to reconstruct population dynamics. (D) The reporter system accurately measured clone abundances, as indicated by strong correlation between barcode abundances in exported and cellular RNA. (E) The reporter system reproducibly measured clone abundances, as indicated by strong correlation of technical replicates. (F) Collective dynamics of drug-resistant and -sensitive populations. Traces show the total abundance of puromycin- and zeocin-resistant cells (purple and green, respectively). CPMS, counts per million of standard. (G) Number of clones detected within each population. (H) Population dynamics of individual clones were resolved by tracking clone barcodes. Relative abundances of 100 randomly selected clones from each population are shown. (I) Distributions of growth rates of clones grown under puromycin (left) or zeocin (right) selection, as determined by fitting an exponential growth equation. Dashed line indicates population-average growth rate determined independently by cell counting. See also Figure S5, Figure S6, and Methods.

Cell populations expand and contract over time. Technologies to track cell population dynamics have advanced our understanding of immune responses^57,58^, viral pathogenesis^59^, tumor growth^60^, and other biological processes. However, existing technologies require either destructive sampling of the analyzed cells, which prevents longitudinal analysis of individual cells, or optical transparency, which is not available in most organisms. As an alternative approach, recovering RNA exported from cells at different timepoints could enable non-destructive tracking of cell populations without optical access. More specifically, if different clones export RNA bearing distinguishable barcodes, then the abundances of these exported barcodes, sampled from cell supernatant, could serve as a proxy for clone abundances to non-destructively resolve their population dynamics (Figure 5A).

To this end, we constructed diverse libraries of exportable barcode sequences and created cells capable of inducible export of these barcodes (Figure 5B). As a base cell line, we stably integrated the exporter Gag-MCP under control of a doxycycline-inducible promoter into HEK293 cells. We designed a lentiviral genetic barcode library comprising a constitutively expressed export-tagged barcode RNA, which includes a 32-nucleotide randomized barcode region and encodes a fluorescent protein reporter. We prepared libraries composed of >5×10^6^ distinct barcodes (Figure S5A) with nearly uniform representation (Figure S5B), providing sufficient diversity to uniquely label >10^4^ cells with <0.2% collision (coincidence) rate (Figure S5C).

To demonstrate monitoring of cell population dynamics, we tracked populations cultured in the presence or absence of growth-inhibiting drugs to which they were either sensitive or resistant (Figure 5C). More specifically, we prepared two polyclonal cell populations, each resistant to either puromycin or zeocin, uniquely labeled cells within these populations using distinct viral barcode libraries, and sorted 5,000 cells per population into the same well. We then cultured these cells for 6 days in the presence of puromycin, zeocin, or neither drug, while inducing barcode RNA export. We collected the culture supernatant daily and counted exported barcodes by sequencing. Using spike-in standards to normalize RNA abundance across samples (Figure S5D), we resolved changes in population abundance over time. Finally, to evaluate the accuracy of the system, we compared the abundances of exported RNA barcodes with their abundances in cellular RNA at the final timepoint.

We first verified that this system accurately and reproducibly reported clone abundances. The majority (63%) of barcodes were detected in both cellular and exported RNA. These barcodes showed strong correlation in their abundances over a >100-fold dynamic range (Pearson r = 0.53) (Figure 5D). Imperfect overlap between the barcodes observed in supernatant and cells consisted almost entirely of barcodes observed in cells but not supernatant. This was partially explained by the spontaneous silencing of RNA exporter expression in 9% of cells during the experiment (Figure S5E). To assess reproducibility, we measured barcode abundances in two replicate aliquots of supernatant from the same well. The majority (84%) of barcodes were detected in both replicates and these showed strong similarity in their abundance (Pearson r = 0.85) (Figure 5E). This imperfect (84%) overlap between replicates arises from technical variation in sequencing library preparation, and establishes a ceiling for the observable overlap between supernatant and cells. Together with silencing, this ceiling explains the imperfect overlap between barcodes detected in supernatant and cellular RNA.

We also characterized the sensitivity of this reporter system, defined by the minimum number of cells of a given clone that can be reliably detected. In a separate experiment, we sorted 10 cells exporting distinct clone barcodes into a single well containing a carrier population of ~30,000 unlabeled HEK293 cells and cultured them for 24 hours to allow accumulation of exported RNA, while limiting cell proliferation to at most a single cell division (Figure S5F). Sequencing exported RNA to saturating depth (Figure S5H) revealed 5.2 ± 3.2 (mean ± s.d.) unique cell barcodes per well (Figure S5G). Because only 64 ± 6% (mean ± 95% c.i.) of cells survived sorting in a control experiment (Methods), these results suggest a lower bound of 81% on single-cell sensitivity at time resolution of one day.

### RNA export enables monitoring of dynamic clonal population responses to drug selection

Monitoring exported RNA barcodes revealed the dynamics of cell populations responding to drug selection. Drug-resistant populations grew exponentially, while sensitive populations declined precipitously (Figure 5F). Within each of the polyclonal resistant and sensitive populations, we resolved thousands of distinct clones (Figure 5G) by sequencing to saturation (Figure S6A), even in the initial sample, in which each clone is represented by only 1 or 2 cells. As drug-resistant populations expanded, additional clones were detected, reflecting the elevated probability of detecting each individual clone as more cells secreted its barcode. By contrast, in drug-sensitive populations, detected clones declined to nearly zero, reflecting clonal extinctions.

Tracking individual clone barcodes revealed the dynamics of thousands of clones (Figure 5H). Examined individually, clones belonging to drug-resistant populations exhibited exponential growth, with a distribution of growth rates whose mean was consistent with values obtained independently by cell counting (Figure 5I and Figure S6B). By contrast, sensitive clones declined in abundance (Figure 5I), often permanently, revealing individual clonal extinction events. Similar patterns of population and clone dynamics were observed with a different growth-inhibiting drug (Figure 5H and Figure 5I). Finally, in the absence of drug selection, both populations grew exponentially, as expected (Figure S6C and Figure S6D).

Taken together, these results show that RNA export enables non-destructive monitoring of mammalian cell population dynamics with clonal resolution and high accuracy, reproducibility, and sensitivity.

### Cell-to-cell delivery of exported RNA

A major challenge in gene and cell therapies is delivery of nucleic acids to specific cell types within an organism. In principle, RNA export could allow engineered sender cells to transfer RNA cargo to non-engineered receiver cells. EPNs have been pseudotyped with viral fusogens, which enable vesicle-cell fusion, to deliver proteins to cells^48^. However, it is unknown whether mRNA can be transferred by EPN-based exporters, such as EPN24-MCP, and expressed in receiver cells at levels sufficient to achieve functional effects.

**Figure 6.**
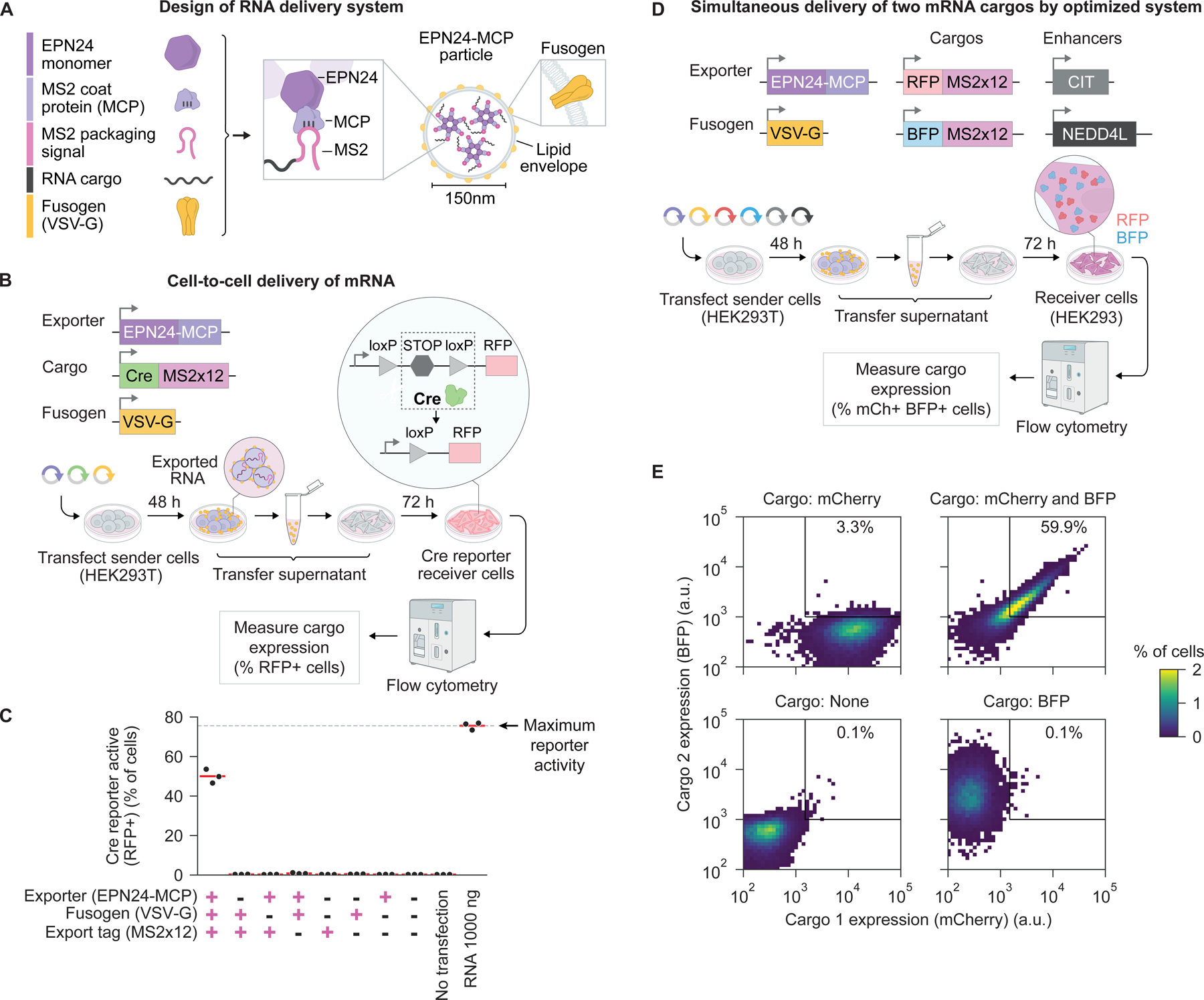
Cell-to-cell delivery and expression of RNA cargo. (A) EPN24-MCP RNA delivery system incorporates a fusogen to enable cell entry and cargo transfer (schematic). (B) HEK293T sender cells were transfected with the delivery system and Cre-expressing mRNA cargo. Conditioned media containing secreted particles was transferred to receiver cells harboring a Cre-activatable RFP cassette. (C) The EPN24-MCP delivery system delivered Cre mRNA cargo to reporter cells. Each dot represents one replicate culture well; solid line indicates mean of replicates. Dashed line indicates maximum activity observed with saturating doses of Cre mRNA transfected into reporter cells. (D) Assay for testing simultaneous dual cargo delivery. (E) Optimized system delivered two mRNA cargos encoding distinct fluorescent proteins to receiver cells (upper right). See also Figure S7.

To test whether pseudotyping enables RNA delivery by EPN24-MCP, we transiently co-expressed a fusogen – the glycoprotein G of vesicular stomatitis virus (VSV-G) – together with the RNA exporter EPN24-MCP and MS2-tagged cargo mRNA in HEK293T cells (Figure 6A), cultured these cells for 48 hours, transferred their filtered supernatant to reporter cells, and then analyzed cargo mRNA expression in the reporter cells (Figure 6B). We first tested a cargo mRNA encoding Cre recombinase, which can permanently activate, through recombination, a red fluorescent protein (RFP) cassette stably integrated in a HEK293 reporter cell line (Methods). With all components present, half of the receiver cell population exhibited reporter activation (50.0 ± 3.5%, mean ± s.d. of 3 replicates) (Figure 6C), compared to a maximum activation rate of 75.6 ± 1.9% achieved by direct transfection of Cre mRNA into reporter cells in a control experiment conducted in parallel (Figure S7A). By contrast, negligible reporter activation was observed when the fusogen, exporter, or export tag on the cargo mRNA were omitted, as expected. By contrast, in similar experiments using the viral RNA exporter Gag-MCP, we observed no RNA delivery (Figure S7B). These results demonstrate RNA can be delivered by pseudotyped EPN24-MCP extracellular vesicles and expressed in receiver cells at functional levels.

RNA delivery efficiency and specificity depended on the expression level of the fusogen (Figure S7C). At low or no VSV-G expression, we observed no Cre recombination in receiver cells. At the highest VSV-G expression levels, we observed promiscuous delivery of RNA even in the absence of the RNA exporter or the export tag, possibly due to VSV-G-driven secretion of extracellular vesicles that lack RNA packaging specificity, as previously reported^61^. Between these extremes, a 20:1 molar ratio of VSV-G expression plasmid to EPN24 expression plasmid optimized delivery efficiency and specificity.

Cellular factors that enhanced RNA export (Figure S2K) also enhanced RNA delivery. Co-expressing CIT or NEDD4L together with the delivery system components enhanced delivery efficiency up to 6-fold (Figure S7D). Based on these results, we used the optimal fusogen expression level and included export enhancers CIT and NEDD4L in subsequent experiments.

To characterize the dynamics of mRNA delivery and resulting protein expression, we monitored mCherry fluorescence over time in receiver cells. Expression was detected as early as 3 hours after transferring supernatant from sender cells to receiver cells (Figure S7E and Figure S7F). The majority of receiver cells (65%) had detectable protein expression by 12 hours after transfer. Given a likely delay of several hours associated with accumulation of fluorescent protein to detectable levels, these results indicate that cargo mRNA enters cells and initiates protein expression within hours after supernatant transfer.

The ability to simultaneously deliver multiple distinct RNA cargos would facilitate transfer of complex genetic programs. We therefore tested delivery of two fluorescent protein mRNA cargos from a single sender cell population (Figure 6D). The optimized delivery system successfully delivered both cargos simultaneously to 60% of receiver cells (Figure 6E). Further, expression of the two cargos was strongly correlated, establishing the potential for delivery of multicomponent RNA circuits.

**Figure 7.**
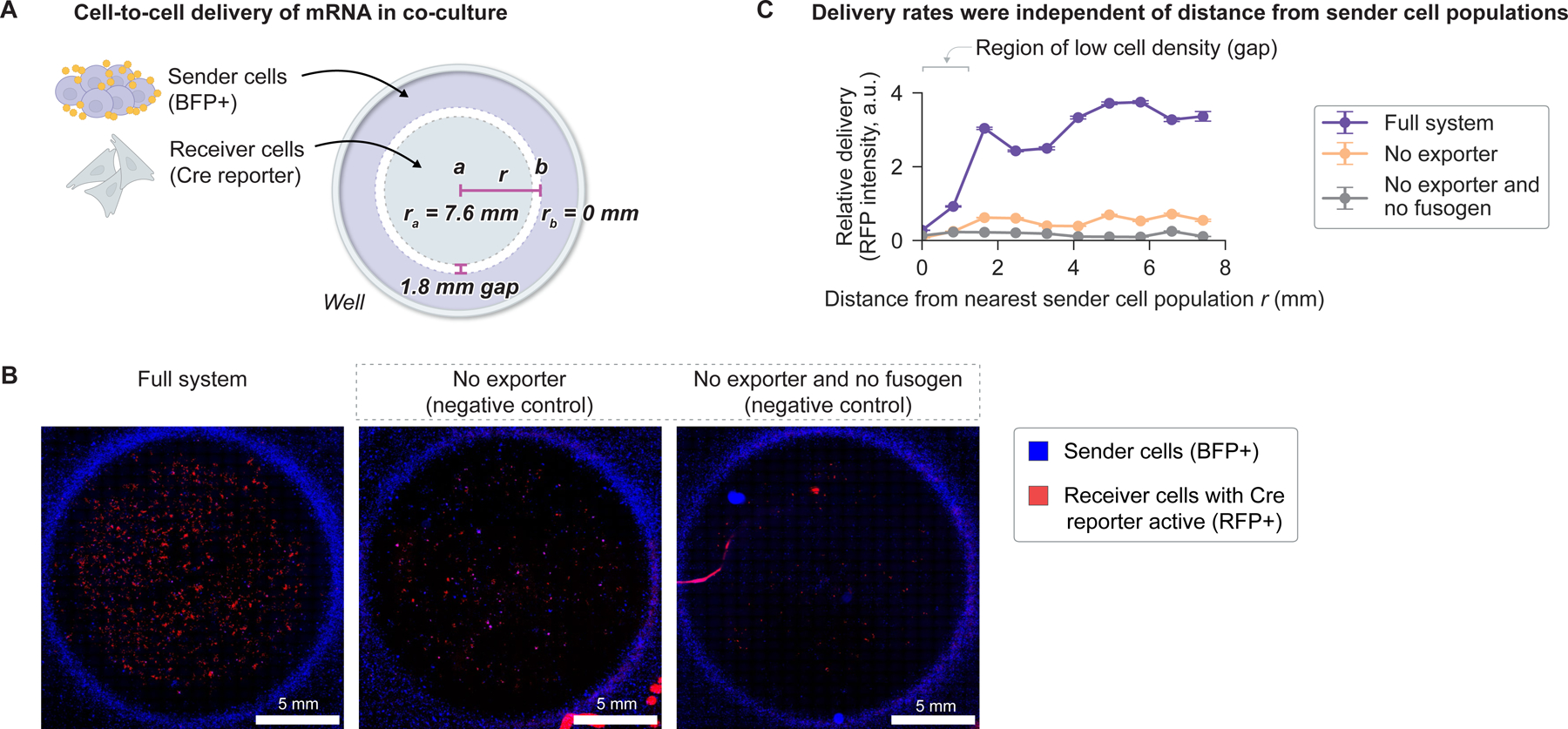
Delivery and expression of RNA in a co-culture setting. (A) To test direct cell-to-cell RNA delivery, we co-cultured sender cells, expressing the optimized delivery system and Cre mRNA cargo, and Cre reporter receiver cells. (B) More delivery was observed throughout the receiver cell population with the full system present, compared to when either the exporter or both exporter and fusogen were omitted. Large pink filaments and blobs are autofluorescent non-cell material. (C) Delivery was independent of the distance from a receiver cell to the nearest sender cell population. Data for (C) are mean and standard deviation of 10 resamplings of pixels within each distance bin.

Finally, we tested whether the EPN24-MCP system is capable of delivering RNA directly from cell to cell in a co-culture setting. Sender cells were transfected with expression plasmids encoding the EPN24-MCP delivery system and a Cre recombinase cargo mRNA. As receiver cells, we used RFP-activating Cre reporter cells (Figure 6B). These sender and receiver cells were co-cultured in distinct spatial regions separated by a 1.8 mm gap, with receivers in the center and senders in the periphery (Figure 7A and Methods). More delivery was observed throughout the receiver population with all system components present, compared to when the fusogen or both fusogen and exporter were omitted (Figure 7B and Figure 7C), as expected. Delivery rates were independent of the distance between a reporter cell and the nearest sender cell population (Figure 7C), consistent with convective transport of delivery particles in culture media. Taken together, these results demonstrate that pseudotyped EPN24-MCP extracellular vesicles enable efficient cell-to-cell delivery of functionally active mRNA.

## Discussion

The ability to export RNA enables monitoring and manipulation of cell behaviors. Because performance in both applications depends on the number of RNA molecules available for detection or expression, export efficiency and specificity are crucial. Through systematic engineering, this study establishes a set of RNA exporters that efficiently and specifically package and secrete target RNA molecules from mammalian cells within protective nanoparticles. For viral capsid-based exporters, we factorized the activities of self-assembly and RNA binding, allowing independent optimization of efficiency and specificity (Figure 1). For EPN-based exporters, we tested modular combinations of functional domains to identify optimal designs (Figure 2). This study also introduces a framework for systematic quantification of RNA export efficiency and specificity, based on RT-qPCR and sequencing, in the fundamental units of molecules per cell per unit time, which should allow benchmarking of current and future export systems.

Protein nanocages provide an ideal foundation for next-generation RNA exporters. Their modularity facilitates engineering, as demonstrated by their robustness to domain permutations and replacements (Figure 2). Alternative nanocage architectures^62,63^ could tune functional properties, such as cargo capacity and stability. Unlike viral proteins^40–46^, nanocage parts are not expected to stimulate antiviral sensing pathways, potentially reducing their propensity for innate immune activation compared to viral capsids, although this remains to be tested experimentally. Importantly for cell-based therapeutic RNA delivery systems, the ability of EPN24-MCP to package target RNA with high specificity reduces risks of toxicity or undesired effects due to transfer of non-target RNA.

RNA export enables molecular information to be obtained non-destructively from living cells. The system described here allows clonally resolved non-destructive monitoring of cell population trajectories with single-cell sensitivity and daily time resolution in complex samples (Figure 5). Efficient, unbiased, non-specific RNA exporters, such as MMLV Gag, also permit non-destructive profiling of cellular transcriptomes (Figure 3). Time resolution is determined by the rates of RNA clearance and the intervals between sample collection. Maximizing export rate and minimizing its cell-to-cell variability should improve time resolution, sensitivity, and accuracy. Importantly, for longitudinal monitoring of cell state dynamics, information about cell state, such as signaling pathway or transcriptional activity, could be encoded by regulating expression of one or more cargo RNAs.

RNA export systems could be adapted for in vivo monitoring of cell populations. Cell-free RNA from nearly every tissue of the human body is detectable in blood^5,64^, and exporters protect RNA from degradation in whole blood (Figure S1N and Figure S2F). Therefore, exporters may enable non-destructive monitoring of cell dynamics *in vivo* by sampling RNA in blood or other fluids. RNA may be most readily detected from cells that interface directly with blood, such as hematopoietic and endothelial cells.

Pseudotyping EPN24-MCP, but not VLP-based, exporters with a fusogen enabled RNA delivery and expression in recipient cells without the need for purification or concentration (Figure 6 and Figure S7B). Because the nanocages used here are porous, RNA cargo may be accessible for translation without requiring their disassembly after fusion, in contrast to many capsids, which must disassemble before RNA translation^65^. Importantly, fusogen-independent cell entry was undetectable, suggesting that further pseudotyping could allow precise targeting of delivery based on cell surface receptor profiles, drawing upon toolkits of natural^66,67^ and engineered fusogens^68–70^. This lack of cell entry in the absence of fusogen also prevents undesirable loss of RNA through cellular uptake in reporter applications.

The EPN24-MCP delivery platform achieved functionally relevant expression levels of fluorescent proteins and Cre recombinase in receiver cells (Figure 6). Delivery of Cre mRNA by EPN24-MCP resulted in recombination in ~50% of receiver cells (Figure 6C), representing an improvement in efficiency over previous VLP-based approaches, which achieved comparable outcomes but required concentration of secreted particles^15,21,22^. Notably, nuclear activity of Cre protein was achieved via mRNA delivery without the need to engineer protein release or localization, in contrast to VLP-based protein delivery^71^. One could similarly transmit RNA encoding secreted protein signals, transcription factors, gene editors, cell death pathways, or more complex circuits to sense and conditionally alter cell states^72^. We anticipate that further development and application of RNA exporters will enable a broad range of biological insights and biomedical approaches.

### Limitations of the study

This study has focused on RNA export from a limited panel of cultured cell lines. Export performance may vary in other cell types and organisms. Properties of secreted particles were characterized only in HEK293 cells after purification by ultracentrifugation with a sucrose cushion, but may vary depending on cell type of origin, growth conditions, or purification process. Cargoes larger than 9.8 kb should be tested to determine the packaging capacity of the EPN24-MCP system. Competition between target and non-target RNA for packaging should be characterized using genome-scale approaches. Although perturbations to cellular physiology due to RNA exporter expression were not detected in this study, we cannot rule out the existence of more subtle perturbations, including potential interactions between natural and engineered RNA secretion pathways. In *in vivo* contexts, the transport of nanoparticles could be impeded by interactions with cells or extracellular matrix components. For delivery, we used the fusogen VSV-G, which enables vesicle-cell fusion, but also can cause cell-cell fusion and cytotoxicity at high expression levels^75,76^. Genetic control of VSV-G expression level or use of alternative, less cytotoxic fusogens^66^ could mitigate these issues. Effects of cell-cell contact on delivery remain to be tested. Finally, in therapeutic applications, the potential immunogenicity of RNA export components must be evaluated and minimized.

## STAR Methods

### Resource availability

#### Lead Contact

Further information and requests for resources and reagents should be directed to and will be fulfilled by the lead contact, Michael B. Elowitz (melowitz@caltech.edu).

#### Materials Availability

Plasmids generated in this study are being submitted to Addgene. All unique/stable reagents generated in this study are available from the lead contact with a completed Materials Transfer Agreement.

#### Data and Code Availability

- Raw DNA and RNA sequencing data have been deposited at the NCBI Sequence Read Archive and are publicly available as of the date of publication. Preprocessed data have been deposited at CaltechDATA and are publicly available as of the date of publication. Accession numbers are listed in the key resources table.
- All original code has been deposited at Github (https://github.com/felixhorns/RNA-export-2023) and is publicly available as of the date of publication. DOIs are listed in the key resources table.
- Any additional information required to reanalyze the data reported in this paper is available from the lead contact upon request.

### Experimental model and study participant details

#### Tissue culture

Cells were cultured under standard conditions. Human embryonic kidney cells (HEK293, HEK293T, and HEK293FT), human lymphoblastoid cells (K562), human T cells (Jurkat), mouse fibroblasts (C3H/10T1/2), and chinese hamster ovary cells (CHO-K1) were cultured in tissue culture-treated plastic plates or flasks at 37 C in humidified chambers with 5% CO2. For HEK293, HEK293T, and HEK293FT cells, growth media consisted of Dulbecco’s Modified Eagle Medium (ThemoFisher), 10% fetal bovine serum (ThermoFisher), 100 units/mL penicillin, 100 μM streptomycin, 2 mM L-glutamine (ThermoFisher), 1 mM sodium pyruvate (ThermoFisher), and 1X Minimal Essential Medium Non-Essential Amino Acids (ThermoFisher). For K562 and Jurkat cells, growth media consisted of Roswell Park Memorial Institute (RPMI) 1640 Medium with GlutaMAX supplement (ThermoFisher), 10% fetal bovine serum (ThermoFisher), 100 units/mL penicillin, 100 μM streptomycin, and 1 mM sodium pyruvate (ThermoFisher). For C3H/10T1/2 cells, growth media consisted of Dulbecco’s Modified Eagle Medium (ThemoFisher), 10% fetal bovine serum (ThermoFisher), 100 units/mL penicillin, and 100 μM streptomycin. For CHO-K1 cells, growth media consisted of Alpha Minimum Essential Medium (Irvine Scientific), 10% fetal bovine serum (ThermoFisher), 100 units/mL penicillin, 100 μM streptomycin, and 2 mM L-glutamine (ThermoFisher). Cells were lifted from plates using 0.05% Trypsin-EDTA (ThermoFisher). Cells were routinely tested with MycoStrip (Invivogen) and confirmed to be negative for mycoplasma.

### Method details

#### Plasmid construction

Constructs used in this study are listed in the Key Resources Table. Some constructs were generated by standard cloning procedures, in which inserts and linearized backbones were generated by polymerase chain reaction (PCR) or restriction digest. The remaining constructs were designed by the authors and synthesized by Genscript. All construct maps are available from CaltechDATA (accession XXX). Selected constructs used for monitoring population dynamics or RNA delivery are deposited at Addgene.

#### Design of nanocage-based RNA exporters

To evaluate structural constraints on design of RNA exporters based on protein nanocages, we examined the design model of the I3–01 protein nanocage^48^ (PDB 5KP9) and an X-ray crystal structure of MS2 coat protein^77^ (PDB 1MSC). Models were displayed and protein geometries were evaluated using PyMol molecular graphics system (2.5.3) (Schrödinger).

#### Particle production and purification

HEK293T cells were plated on 10 cm dishes with 6,000,000 cells per dish, and co-transfected the following day with 10 ug of RNA exporter plasmid and 10 ug of reporter plasmid using calcium phosphate. Media was harvested 48 hours after transfection. Exporter particles were purified and concentrated approximately 500-fold by ultracentrifugation in a cushion of 20% (w/v) sucrose in phosphate buffered saline (PBS). Particles from all export systems pelleted below the 20% sucrose, indicating that they have buoyant densities < 1.08 g/cm^3^.

#### Electron microscopy

For electron microscopy, as shown in Figure 1, Figure 2 and Figure S1B, purified supernatant was adsorbed onto freshly glow-discharged PureC 300 mesh carbon-coated copper grids (Ted Pella) for one minute followed by staining with 1.5% uranyl formate (Electron Microscopy Sciences) for another minute. Grids were imaged using Talos Arctica (ThermoFisher) equipped with Gatan K3 Summit direct electron detector at 200 keV and nominal magnification of 28000X (1.44 Å/pixel) using SerialEM^79^ and Digital Micrograph software.

#### Dynamic light scattering

For dynamic light scattering (DLS), as shown in Figure S1 and Figure S2, purified supernatant was diluted 200-fold in Dulbecco’s Phosphate Buffered Saline containing calcium chloride and magnesium chloride (ThermoFisher), passed through a 0.45 μm cellulose acetate syringe filter (VWR), and 100 μL of this sample was added to a UVette 220–1600 nm cuvette (Eppendorf). DLS measurements were performed using a Wyatt DynaPro NanoStar instrument with a 658 nm laser in batch mode with twenty acquisitions at 25 ± 0.1 C and an angle of 90°. Data were evaluated using the DYNAMICS software (Wyatt Technologies).

#### Reverse transcription and quantitative PCR

We used an RT-qPCR assay to measure the abundance of specific RNA molecules in exported or cellular RNA. For exported RNA, RNA was extracted from 140 μL of supernatant using the Viral RNA Mini kit (Qiagen) according to manufacturer’s instructions with inclusion of carrier RNA. For cellular RNA, RNA was extracted from cells using the RNeasy Mini kit (Qiagen) according to manufacturer’s instructions with inclusion of 2-mercaptoethanol. RNA was treated with Turbo DNase (ThermoFisher) at 37 C for 30 min according to manufacturer’s instructions, including the use of inactivation and cation removal reagents. RNA was then reverse transcribed using iScript Reverse Transcription Supermix (Bio-Rad) at 25 C for 5 minutes, 46 C for 20 minutes, and 95 C for 1 minute. Typically, 10 μL of RNA was used as input (corresponding to ~500 ng of total RNA). Quantitative polymerase chain reaction (qPCR) was performed on the CFX96 Touch system (Biorad) using iQ SYBR Green Supermix (Biorad) with 1 μL of reverse transcription product as input, final concentration of 300 nM per primer, and thermal cycling profile consisting of 95 C for 3 minutes, followed by 40 cycles of 95 C for 10 seconds and 67 C for 30 seconds. Primer sequences are listed in Key Resources Table. For mCherry, primers oFH77 and oFH78 were used. For Cre, primers oFH189 and oFH190 were used with an annealing temperature of 63 C instead of 67 C. Each sample was measured in triplicate and quantified based on a standard curve of expression plasmid of the target gene using the CFX Maestro software (Biorad). Lower limits of quantification were calculated based on measured RT-qPCR signal in negative controls consisting of supernatant from HEK293T cells subjected to mock transfections without DNA, or alternatively, if there was no signal in any such negative controls of a given experiment, then based on the expected signal from a single molecule of input cDNA to the qPCR reaction, accounting for the efficiency of the protocol. To confirm that this RT-qPCR assay faithfully measured RNA, as shown in Figure S1D, rather than potential contaminants such as transfected DNA expression plasmids, the same procedure was carried out, except omitting reverse transcription.

To determine the overall efficiency of this protocol, we used an *in vitro* transcribed mCherry mRNA standard. The production of this RNA is described below. RNA was quantified using the Qubit RNA HS Assay kit (Fisher) and full-length product was confirmed using the RNA 6000 Pico kit with the Bioanalyzer instrument (Agilent). For determining efficiency, RNA was added to nuclease-free water and its abundance was measured using the full RT-qPCR protocol described above (starting with RNA extraction). Based on the measured abundance and the independently measured amount of input RNA (based on Qubit), we calculated the overall detection efficiency of the protocol to be 5.96 × 10^−3^ and this value was used to determine RNA abundance in input samples.

#### Producing in vitro transcribed mRNA standards

We used mRNA standards to determine the efficiency of the RT-qPCR protocol; determine the relative abundance of clone barcode RNA by normalization to spike-in standards; and validate the RNase protection assay. These mRNA standards were produced by *in vitro* transcription as follows. Linear DNA for *in vitro* transcription was generated using PCR, simultaneously adding a 5’ T7 RNA polymerase promoter followed by AG dinucleotide and a 3’ 120-nucleotide poly-adenosine tract. The PCR product was purified using the DNA Clean and Concentrator-5 kit (Zymo). mRNA synthesis was carried out using the HiScribe T7 High Yield RNA Synthesis kit (NEB) with 500 ng of linear DNA template, 5 mM each of ATP, GTP, CTP, and N1-Methyl-Pseudouridine-5’-Triphosphate (TriLink), and 4 mM CleanCap (TriLink). The reaction was incubated at 37 C for 2 hours, followed by 15 minute DNase treatment, and finally RNA was purified using the RNA Clean and Concentrator-5 kit (Zymo). RNA was further treated with Turbo DNase (ThermoFisher) and purified again using the RNA Clean and Concentrator-5 kit (Zymo).

#### Measuring RNA loss due to cleanup steps

To determine the loss of RNA due to the cleanup steps of clarification and filtration, as shown in Figure S1C, HEK293T cells were plated on 12-well plates with 200,000 cells per well. Cells were co-transfected the following day with 1000 ng RNA exporter Gag-MCP plasmid and 1000 ng reporter mCherry-MS2×12 plasmid using Lipofectamine 3000 (ThermoFisher) according to manufacturer’s instructions. Media was replaced with 1 mL of fresh media at 8 hours after transfection. Media was harvested 48 hours after transfection. Separate aliquots of the same media were subjected to clarification by centrifugation at 3000 g for 5 minutes, filtration through a cellulose acetate filter with 0.45 μm pore size (VWR), or both clarification and filtration. Reporter RNA abundance was measured using RT-qPCR. Similar results were obtained for the RNA exporter EPN24-MCP, for which the cleanup steps of clarification and filtration reduced RNA recovery by 20% and 71% respectively, leading to a combined loss of 77%.

#### Measuring export by viral RNA exporters

To measure the efficiency and specificity of RNA export by viral RNA exporters, as shown in Figure 1 and Figure S1, HEK293T cells were plated on 12-well plates with 200,000 cells per well. Cells were co-transfected the following day with 1250 ng RNA exporter plasmid and 1250 ng reporter plasmid using Lipofectamine 3000 (ThermoFisher) according to manufacturer’s instructions. Media was replaced with 1 mL of fresh media at 8 hours after transfection. Media was harvested 48 hours after transfection, clarified by centrifugation at 3000 g for 5 minutes, passed through a cellulose acetate filter with 0.45 μm pore size (VWR), and reporter RNA abundance was measured using RT-qPCR. The results of this screen are shown in Figure S1K. To confirm that target RNA was expressed consistently in cells across samples, as shown in Figure S1L, RNA was extracted from cells in the same experiment at 48 hours after transfection and reporter RNA abundance was measured using RT-qPCR. These results were reproduced by transfecting cells in 24-well plates with 500 ng RNA exporter plasmid and 500 ng reporter plasmid, as shown in Figure 1.

#### Measuring export by nanocage-based exporters

To measure the efficiency and specificity of RNA export by protein nanocage-based RNA exporters, as shown in Figure 2, we used HEK293T reporter cell lines with stable constitutive expression of mCherry with or without MS2 export tags (specifically, 8 MS2 aptamer export tags in a repetitive array in the 3’ UTR, denoted MS2×8) (denoted cFH16 and cFH15, respectively). Construction of these cell lines is described below. Reporter cells were transfected in 96-well plates, as described above for viral RNA exporters, except with 50 ng of RNA exporter construct, and exported RNA was measured by RT-qPCR.

#### Measuring rate of RNA export

To measure the rate of RNA export, as shown in Figure S1E, HEK293T cells were plated on 24-well plates with 168,000 cells per well. Cells were co-transfected the following day with 500 ng of RNA exporter plasmid and 500 ng of mCherry-MS2×8 reporter plasmid using Lipofectamine 3000 (ThermoFisher) according to manufacturer’s instructions. At 24 hours after transfection, media was removed, cells were washed with 1 mL of fresh media, and finally 1 mL of fresh media was added. Media was then collected at timepoints of 0, 18, and 24 hours after this step, clarified by centrifugation at 3000 g for 5 minutes, passed through a cellulose acetate filter with 0.45 μm pore size (VWR), and reporter RNA abundance was determined using RT-qPCR in triplicate. To count cells, 200 μL of Trypsin-EDTA (ThermoFisher) was added to the well after media was collected and incubated at 37 C for 5 minutes. Cell counts were determined using the Countess 3 automated cell counter (ThermoFisher). Microscopy confirmed that no cells remained attached to the plate. Transfection efficiency was determined using the CytoFLEX S flow cytometer (Beckman Coulter) based on expression of fluorescent markers and used to calculate the number of cells expressing both exporter and reporter. We fit a linear model by least squares regression and the rate of export was determined based on the slope with variance determined by error propagation.

#### Dependence of export on export tag number

To characterize how the RNA export rate of Gag-MCP depends on the copy number of the MS2 export tag, as shown in Figure S1F, HEK293T cells were transfected in 12-well plates with 1250 ng of RNA exporter Gag-MCP plasmid and 1250 ng of mCherry reporter plasmid with varying numbers of MS2 aptamer export tags in a repetitive array in the 3’ UTR, then RT-qPCR was used to measure reporter RNA abundance in supernatant at 48 hours after transfection. Similarly, to characterize how the RNA export rate of EPN24-MCP depends on the copy number of the MS2 export tag, as shown in Figure S2B, HEK293T cells were transfected in 48-well plates with 75 ng of RNA exporter EPN24-MCP plasmid and 250 ng of mCherry reporter plasmid with varying numbers of MS2 aptamer export tags in a repetitive array in the 3’ UTR, then RT-qPCR was used to measure reporter RNA abundance in supernatant at 48 hours after transfection.

#### Dependence of export on component expression

To characterize how the RNA export rate of Gag-MCP depends on the expression levels of the exporter and reporter RNA, as shown in Figure S1G and Figure S1H, HEK293T cells were transfected in 48-well plates with 250, 75, or 25 ng of RNA exporter Gag-MCP plasmid and 250, 75, or 25 ng of mCherry reporter plasmid (corresponding to the 1X, 0.3X, and 0.1X amounts of plasmid, respectively). RT-qPCR was used to measure reporter RNA abundance in supernatant at 48 hours after transfection.

#### Export by genomically integrated transgenes

To test RNA export using expression of components from stable genomically integrated transgenes, as shown in Figure S1I, we used the cell lines cFH38, which expresses the RNA exporter Gag-MCP and the reporter RNA mCherry-MS2×8, and cFH16, which expresses only the reporter RNA mCherry-MS2×8 (see “Cell line construction”). Cells were plated in a 48-well plate in media containing 1 ng/μL doxycycline hydrochloride to induce RNA exporter expression (Sigma). Media was changed after 24 hours, collected after an additional 48 hours, then passed through a cellulose acetate filter with 0.45 μm pore size (VWR). RT-qPCR was used to measure reporter RNA abundance in supernatant. Because cultures could contain a mixture of expressing and non-expressing cells, this assay produced a lower bound on the efficiency of RNA export.

#### RNase protection assay

To characterize protection from RNase challenge, as shown in Figure 1L and Figure 2F, HEK293T cells were plated on 24-well plates with 168,000 cells per well. Cells were co-transfected the following day with 500 ng of RNA exporter plasmid and 500 ng of mCherry reporter plasmid containing the appropriate export tag for the exporter (MS2×8 or Psi) using Lipofectamine 3000 (ThermoFisher) according to manufacturer’s instructions. Media was replaced with 1 mL of fresh media at 8 hours after transfection. Media was harvested at 72 hours after transfection, clarified by centrifugation at 3000 g for 5 minutes, passed through a cellulose acetate filter with 0.45 μm pore size (VWR), and used fresh in assays (without freezing). RNase challenge was performed in a total volume of 140 μL containing 28 μL of media, 2 mM EDTA, 312.5 mM NaCl, and 50 mM Tris-HCl pH 7.5. Conditions with RNase contained 0.4 ug/mL RNase A (ThermoFisher), and 1 U/mL RNase T1 (ThermoFisher). Conditions with detergent contained 0.1% Triton X-100 (Fisher Scientific). Samples were prepared on ice, incubated at 37 C for 15 minutes, returned to ice, and 20 U of RNase inhibitor SUPERase-In (ThermoFisher) was added. RNA was immediately extracted using the Viral RNA Mini kit (Qiagen) and DNase treatment and RT-qPCR were performed as described above. To validate the assay, *in vitro* transcribed mCherry mRNA, which was not packaged and therefore not expected to be protected from RNase activity, was diluted in H2O and used as input in place of media. Protection from RNase degradation was determined using samples lacking RNase and detergent as a reference.

#### Stability of exported RNA in supernatant

To characterize the stability of RNA encapsulated by Gag-MCP or EPN24-MCP during incubation in cell culture supernatant, as shown in Figure S1M and Figure S2E, HEK293T cells were plated on a 12-well plate with 200,000 cells per well. Cells were co-transfected the following day with 1000 ng of Gag-MCP plasmid or 300 ng of EPN24-MCP plasmid and 500 ng of mCherry-MS2×12 reporter plasmid using Lipofectamine 3000 (ThermoFisher) according to manufacturer’s instructions. Media was replaced with 1 mL of fresh media at 8 hours after transfection. Media was harvested at 48 hours after transfection, clarified by centrifugation at 3000 g for 5 minutes, passed through a cellulose acetate filter with 0.45 μm pore size (VWR), and used fresh in assays (without freezing). 400 μL aliquots of the supernatant were prepared in separate tubes, incubated at 37 C in a humidified chamber with 5% CO2 for 0, 1, 2, 4, or 6 days, then stored at −80 C until further processing. RT-qPCR was used to measure reporter RNA abundance after incubation. Stability was determined using the samples stored immediately at −80 C (i.e., not incubated) as a reference. Given that disruption of VLPs by detergent led to rapid degradation of the encapsulated RNA (Figure 2F), the stability of exported RNA in supernatant was likely dominated by the rate of particle degradation, rather than the rate of RNA degradation.

#### Stability of exported RNA in whole blood

To characterize the stability of RNA encapsulated by Gag-MCP or EPN24-MCP during incubation in whole blood, as shown in Figure S1N and Figure S2F, HEK293T cells were plated on 10 cm dishes with 6,000,000 cells per dish, and co-transfected the following day with 10 ug of RNA exporter plasmid and 10 ug of reporter plasmid using calcium phosphate. Media was harvested 48 hours after transfection. Exporter particles were purified and concentrated approximately 500-fold by ultracentrifugation in a cushion of 20% (w/v) sucrose in phosphate buffered saline (PBS), and stored at −80 C until further use. Purified particles were thawed and diluted 10-fold in DPBS without calcium and magnesium. 5 μL of this diluted sample was combined with 95 μL of whole CD1 mouse blood (Innovative Research), incubated at 37 C for 0 minutes, 15 minutes, 1 hour, 4 hours, or 24 hours, then transferred to ice. RNA was extracted immediately using the Zymo Whole Blood RNA kit (Zymo). RT-qPCR was used to measure reporter RNA abundance. To validate the assay, *in vitro* transcribed mCherry mRNA, which was not packaged and therefore not expected to be protected from degradation, was diluted in H2O and used as input. Stability was determined using the samples extracted immediately (i.e., not incubated) as a reference. Note that variability in the yield of RNA purification likely explains the apparent recovery of >100% of RNA after incubation.

#### Rate of RNA export by EPN24-MCP

To compare the rate of RNA export by EPN24-MCP to that of Gag-MCP, as shown in Figure S2A, we measured the amount of RNA accumulated in supernatant by each exporter after either 48 or 72 hours. For the 48 hour measurement, HEK293T cells were transfected in 48-well plates with 250 ng of Gag-MCP plasmid or 75 ng of EPN24-MCP plasmid and 250 ng of mCherry-MS2×12 reporter plasmid. For the 72 hour measurement, HEK293T cells were transfected in 24-well plates with 500 ng of Gag-MCP plasmid or 150 ng of EPN24-MCP plasmid and 500 ng of mCherry-MS2×12 reporter plasmid. In both cases, media was replaced with 1 mL of fresh media at 8 hours after transfection. Media was harvested at either 48 or 72 hours after transfection, clarified by centrifugation at 3000 g for 5 minutes, and passed through a cellulose acetate filter with 0.45 μm pore size (VWR). RT-qPCR was used to measure reporter RNA abundance in supernatant. Because the samples at the 48 and 72 hour timepoints were prepared in separate experiments, their abundances should not be directly compared with each other for purposes of extracting export rates. Nonetheless, the results show that the amounts of RNA exported by Gag-MCP and EPN24-MCP are comparable across multiple timepoints.

#### RNA cargo capacity of EPN24-MCP

To estimate the cargo capacity of EPN24-MCP, we measured the dependence of export efficiency on cargo RNA length. We designed and expressed transcripts of increasing length by introducing non-coding sequences into the 3’ UTR of a cargo mRNA encoding the Cre recombinase. The abundances of cargo RNA within exporting cells (Figure S2G) and in culture supernatant (Figure S2H) both declined with length, but their ratio, which is a measure of export efficiency, remained within a 3-fold range up to the longest cargo length tested, ~9.8kb (Figure S2I). This result suggests that export cargo capacity reaches at least 9.8kb.

More specifically, to characterize how export rates depend on the length of RNA cargo, as shown in Figure S2G, Figure S2H, and Figure S2I, expression plasmids for RNA cargos of varying length were constructed by cloning non-coding sequences into the 3’ UTR of a Cre-MS2×12 expression plasmid between the MS2 export tag array and the poly-A signal sequence. HEK293T cells were transfected in 12-well plates with 375 ng of RNA exporter EPN24-MCP plasmid and 1250 ng of Cre-MS2×12 cargo RNA plasmid of varying lengths. Media was replaced with 1 mL of fresh media at 8 hours after transfection. Media was harvested at 48 hours after transfection, clarified by centrifugation at 3000 g for 5 minutes, and passed through a cellulose acetate filter with 0.45 μm pore size (VWR). RT-qPCR was used to measure cargo RNA abundance in cells, as shown in Figure S2G, and supernatant, as shown in Figure S2H. The ratio of RNA abundances in supernatant and cells was used as a metric of export efficiency that accounts for the availability of cargo RNA within exporting cells for packaging and secretion, as shown in Figure S2I. PCR with primers targeting the 3’ end of the cargo transcript confirmed that full-length mRNA cargo was exported.

#### RNA export activity of EPN24-PCP

To test the compatibility of nanocage-based RNA exporters with alternative RNA binding proteins, as shown in Figure S2M, we substituted the sequence-specific RNA binding domain PP7 bacteriophage coat protein (PCP), which binds specifically to the PP7 RNA hairpin aptamer^53^, in place of MCP, forming EPN24-PCP. HEK293T cells were transfected in 12-well plates with 375 ng of RNA exporter EPN24-MCP or EPN24-PCP plasmid and 1250 ng of mCherry reporter plasmid with either MS2 or PP7 aptamer export tags or no export tag, then RT-qPCR was used to measure reporter RNA abundance in supernatant at 48 hours after transfection. These EPN24-PCP constructs exported target RNA containing the PP7 aptamer export tag with efficiency comparable to that with which EPN24-MCP exported MS2-containing transcripts, albeit lower specificity (Figure S2M). The two RNA binding domains were orthogonal, as EPN24-PCP did not export target RNA containing the MS2 aptamer export tag and vice versa. Thus, nanocage-based RNA exporters can support modular engineering of RNA targeting specificity to create multiple export channels.

#### Expressing cellular factors to enhance export

For testing whether overexpression of cellular factors can enhance RNA export, as shown in Figure S2J and Figure S2K, we designed expression plasmids of six candidate modulators. For NEDD4L, we used the naturally occurring ΔC2 isoform (GenBank AAM46208), denoted NEDD4LΔC2 which enhances budding of HIV particles from human cells^49,51^. We used full-length isoforms of CIT (Uniprot O14578–1) and UGCG (Uniprot Q16739) that also enhance release of HIV particles^51^. We used isoforms of STEAP3 (DNASU HsCD00081929) and SDC4 (DNASU HsCD00074146) that enhance production of exosomes^24^. For the gap junction protein Cx43 (DNASU HsCD00434989), we used a constitutively active mutant (S368A) that enhances transfer of exosomal contents to receiver cells^24^. HEK293T cells were transfected in 12-well plates with 375 ng of RNA exporter EPN24-MCP, 1250 ng of mCherry reporter plasmid, and 1250 ng of candidate export modulator expression plasmid, then RT-qPCR was used to measure reporter RNA abundance in supernatant at 48 hours after transfection.

#### Suppressing export using ESCRT inhibitor

To test whether overexpression of a dominant negative inhibitor of the ESCRT pathway affects RNA export, as shown in Figure S2L, HEK293T cells were transfected in 12-well plates with 375 ng of RNA exporter EPN24-MCP, 1250 ng of mCherry reporter plasmid, and 625 ng of VPS4-E228Q expression plasmid, then RT-qPCR was used to measure reporter RNA abundance in supernatant at 48 hours after transfection.

#### Measuring export of non-target RNA by RT-qPCR

In principle, target RNA could compete with non-target RNA for export. We therefore examined the dependence of non-target RNA export on the presence and abundance of target RNA. We focused on a representative non-target mRNA, GAPDH, which is highly expressed in HEK293T cells. The abundance of exported GAPDH mRNA, as measured by RT-qPCR, reflected the specificity of RNA export systems, as measured by genome-scale RNA sequencing. In the supernatant of cells expressing MMLV Gag, but not its target RNA, GAPDH mRNA was present at elevated abundance (ΔCq = 10.5 ± 0.1, mean ± s.d. of 3 replicates) compared with cells expressing MMLV Gag together with its target RNA mCherry-Psi (ΔCq = 9.6 ± 0.2, mean ± s.d. of 3 replicates) (ΔΔCq = 0.9, P = 0.04, Mann-Whitney U test, two-sided) (Figure S3D). Similarly, in the supernatant of cells expressing Gag-MCP together with varying amounts of its target RNA mCherry-MS2×12, GAPDH mRNA was not detectable with the highest amount of target RNA expression, but slightly elevated export of GAPDH mRNA was evident with both 3- and 10-fold reduction of the target RNA expression plasmid amount (Figure S3E). These results suggest that target and non-target RNA compete for packaging in MMLV Gag and Gag-MCP VLPs.

To test export of non-target GAPDH mRNA by MMLV Gag, as shown in Figure S3D, HEK293T cells were transfected in 24-well plates with 500 ng of MMLV Gag plasmid and 500 ng of mCherry-MS2×12 reporter plasmid. Media was replaced with 1 mL of fresh media at 8 hours after transfection. Media was harvested at 72 hours after transfection, clarified by centrifugation at 3000 g for 5 minutes, and passed through a cellulose acetate filter with 0.45 μm pore size (VWR). To test export of non-target GAPDH mRNA by Gag-MCP, as shown in Figure S3E, HEK293T cells were transfected in 48-well plates with 250 ng of Gag-MCP plasmid and 250, 75, or 25 ng of mCherry-MS2×12 reporter plasmid (corresponding to 1X, 0.3X, and 0.1X amount of target RNA plasmid, respectively). Media was replaced with 1 mL of fresh media at 8 hours after transfection. Media was harvested at 48 hours after transfection, clarified by centrifugation at 3000 g for 5 minutes, and passed through a cellulose acetate filter with 0.45 μm pore size (VWR). In both cases, RT-qPCR was used to measure GAPDH mRNA abundance in supernatant with oFH57 and oFH58 as primers and an annealing temperature of 63 C.

#### Export from K562 and Jurkat cells

To test export of RNA from human blood cell lines, as shown in Figure 4A and Figure 4B, K562 and Jurkat cells (200,000 cells per condition) were electroporated with 500 ng of RNA exporter plasmid and 500 ng of mCherry-MS2×8 plasmid using a 4D-Nucleofector (Lonza) in the 16-well Nucleocuvette Strip format according to manufacturer’s instructions, using SF Cell Line reagents for K562 cells and SE Cell Line reagents for Jurkat cells. After electroporation, 80 μL of media was added to electroporation wells and cells were incubated for 10 minutes at 37 C. Jurkat cells were plated in a 96-well plate in a final volume of 200 μL. K562 cells were plated in a 48-well plate in a final volume of 300 μL. Media was harvested 48 hours after electroporation, cells were pelleted by centrifugation at 150 g for 7 minutes, and supernatant was clarified by centrifugation at 3000 g for 5 minutes. Reporter RNA abundance was measured using RT-qPCR.

#### Export from C3H/10T1/2 and CHO-K1 cells

To test export of RNA from rodent cell lines, as shown in Figure 4C and Figure 4D, C3H/10T1/2 and CHO-K1 cells were plated in 24-well plates with 100,000 cells per well. Cells were co-transfected the following day with 500 ng Gag-MCP plasmid or 350 ng EPN24-MCP plasmid and 500 ng mCherry-MS2×8 plasmid using Lipofectamine 3000 (ThermoFisher) according to manufacturer’s instructions. Media was replaced with 1 mL of fresh media at 8 hours after transfection. Media was harvested at 48 hours after transfection, clarified by centrifugation at 3000 g for 5 minutes, and stored at −80 C. Reporter RNA abundance was measured using RT-qPCR. RNA abundances were scaled by the number of cells expressing the RNA export system components, as determined by flow cytometry and cell counting.

#### Characterizing RNA export by sequencing

##### Sample preparation and sequencing

To characterize RNA export by sequencing, as shown in Figure 3 and Figure S3, HEK293T cells were plated on 12-well plates with 300,000 cells per well. Cells were co-transfected the following day with 1250 ng of RNA exporter plasmid (375 ng in the case of EPN24-MCP), 1250 ng of mCherry-Psi or mCherry-MS2×8 export tag-containing reporter plasmid, and 1250 ng of mTagBFP2 reporter plasmid without export tag using Lipofectamine 3000 (ThermoFisher) according to manufacturer’s instructions. Media was replaced with 1 mL of fresh media at 8 hours after transfection. Media was harvested at 48 hours after transfection, clarified by centrifugation at 3000 g for 5 minutes, passed through a cellulose acetate filter with 0.45 μm pore size (VWR), and stored at −80 C.

To sequence exported RNA, External RNA Controls Consortium (ERCC) synthetic spike-in RNAs (ThermoFisher) were diluted 1:200 in nuclease-free water and 1 μL was added per 560 μL of Buffer AVL (Qiagen). RNA was extracted using the Viral RNA Mini kit (Qiagen) according to manufacturer’s instructions with 280 μL of supernatant as input. RNA was treated with Turbo DNase (ThermoFisher) and purified using the RNA Clean and Concentrator-5 kit (Zymo). Sequencing libraries was prepared using the SMARTer Stranded Total RNA-Seq v2 Pico Input kit (Takara) with 3–15 ng of RNA input (fixed volume of 7 μL of input RNA), no fragmentation step, and 16 cycles of amplification by PCR. Libraries were sequenced by Novogene using the NovaSeq 6000 platform (Illumina) with 20–30M paired-end 150 bp reads per sample.

To sequence cellular RNA, after media collection, cells were harvested by adding 350 μL of ice-cold Buffer RLT (Qiagen) containing 2-mercaptoethanol directly to the well, scraping the well with a pipette tip, and storing the solution at −80 C. RNA was extracted using the RNeasy Mini kit (Qiagen), treated with TurboDNase (ThermoFisher), and purified using RNA Clean and Concentrator-5 kit (Zymo). mRNA sequencing libraries were prepared by NovoGene. Briefly, mRNA was purified from total RNA using poly-T oligo-attached magnetic beads, then libraries were prepared using the NEBNext Ultra II RNA kit for Illumina (NEB), which uses random hexamer priming, and sequenced using the NovaSeq 6000 platform (Illumina) with 20–30M paired-end 150 bp reads per sample.

##### Preprocessing of sequencing data

Reads from both exported and cellular RNA sequencing were aligned to a custom reference genome using STAR (2.7.8a)^80^ with the ENCODE standard options except “--outFilterScoreMinOverLread 0.3 --outFilterMatchNminOverLread 0.3 –outFilterMismatchNmax 20 --outFilterMismatchNoverLmax 0.3 --alignSJoverhangMin 5 --alignSJDBoverhangMin 3”. This reference consisted of the human genome GRCh38.103 with ERCCs (https://assets.thermofisher.com/TFS-Assets/LSG/manuals/ERCC92.zip) and EGFP, mCherry, and mTagBFP2 coding sequences. Uniquely mapped reads that overlap with genes were counted using HTSeq-count (0.13.5)^81^ with default settings except “-m intersection-strict”. To normalize for differences in sequencing depth across samples, we rescaled gene counts to counts per million (CPM).

To normalize the abundance of extracellular RNA across supernatant samples using the ERCC spike-in standards, we used the mock transfected sample as a reference. For each ERCC transcript (92 in total), we calculated the ratio of its abundance in each sample to its abundance in the reference sample. We then calculated the geometric mean of this ratio across the 63 transcripts having mean abundance >10 CPM. We normalized gene abundances by dividing all counts in each sample by this ratio (in CPM space), achieving equivalent mean abundance of these ERCCs across all samples. We refer to this normalized abundance as counts per million of standard (CPMS).

##### Analyzing export efficiency, specificity, and bias

Abundance and enrichment of endogenous transcripts in supernatant, as shown in Figure 3B and Figure S3B, was visualized by plotting the abundance (normalized using ERCCs) in the presence of the exporter versus a matched sample without the exporter (transfected only with expression plasmids for mCherry with the appropriate export tag and mTagBFP2 without export tag). Comparison of relative abundance in cells versus supernatant, as shown in Figure 3E, was performed using counts per million reads (CPM) without ERCC normalization (because the relative abundance of transcripts within each sample is independent of between-sample normalization).

PANTHER^82^ was used to analyze pathway enrichment among transcripts overrepresented in supernatant compared with cells (defined as having expression levels exceeding 9 CPM in cells and enrichment of at least 16-fold in supernatant versus cells). Overrepresentations of GO terms among these enriched genes were only marginally significant and inconsistent across exporters. For example, the most significant overrepresentations were miRNA-mediated gene silencing (false discovery rate, FDR = 0.024), macromolecule metabolic process (FDR = 0.046), and trans-Golgi network transport vesicle (FDR = 0.023) for MMLV Gag, Gag-MCP, and EPN24-MCP, respectively. Similar results were obtained with varying expression level and enrichment cutoffs.

##### Detection rate of endogenous transcripts

This ability to export unbiased samples of the cytoplasmic transcriptome could enable non-destructive genome-scale monitoring of cell states. To assess the information content of exported endogenous RNA, we examined the detection rate of transcripts in exported RNA as a function of their cellular expression level. To assess the detection rate of endogenous transcripts in supernatant, genes were binned according to their expression level in the cellular transcriptome. Within each bin, the rate of detection in supernatant was calculated as the fraction of genes in the bin that were present at levels exceeding 1 CPM in supernatant. These rates are shown as a function of the mean expression level within the bin in Figure S3C. Using MMLV Gag (the exporter which exports non-target RNA most efficiently), expressed transcripts were detected in exported RNA with substantially greater sensitivity compared to without an exporter, achieving detection rates of >50% for all genes expressed at counts per million (CPM) ≥ 3 (Figure S3C). These transcripts included markers of cell identity, such as the epithelial marker Desmoglein-2 and the mesenchymal markers N-Cadherin and Vimentin, demonstrating that exported RNA contained sufficient information to identify the mixed epithelial-mesenchymal state of HEK293T cells^55^. By contrast, engineered viral and nanocage-based exporters, which were more specific for their target RNA, yielded lower detection rates of cellular transcripts, as expected (Figure S3C). Together, these results show that engineered RNA export enables non-destructive, genome-wide monitoring of transcriptional states at the cell population level.

##### Contribution of endogenous export sources

Total cell-free RNA (in supernatant) includes RNA from endogenous extracellular vesicles (EVs), as well as lysed cells and other natural sources of export. In control samples lacking exporters, cell-free RNA was detected in supernatant, which reflects the contribution of these endogenous export sources. In samples where RNA exporters were expressed, reporter transcripts were detected at much higher abundance in comparison with the control samples, indicating that export via engineered RNA export systems occurs at much higher rates than via endogenous pathways. We quantified off-target export by comparing samples having exporter versus negative control samples lacking exporter. Because both of these types of samples include endogenous EVs, any differences detected in this comparison cannot be explained by endogenous EVs, unless these EVs were induced by the engineered RNA export system itself.

##### Differential expression of cellular RNA

To characterize alterations to the cellular transcriptome due to RNA exporter expression, as shown in Figure S4D, differential expression analysis was performed using DESeq2 (1.30.1)^83^ in R (4.0.5) comparing raw gene counts in cells transfected with and without RNA exporters.

#### Morphology of cells transiently exporting RNA

To image cells transiently expressing RNA exporters, as shown in Figure S4A, HEK293T cells were plated on 24-well glass-bottom plates (MatTek) with 168,000 cells per well after coating the well with Poly-D-Lysine (ThermoFisher). Cells were co-transfected the following day with 500 ng of RNA exporter plasmid (150 ng in the case of EPN24-MCP) and 500 ng of reporter plasmid (either mCherry-Psi or mCherry-MS2×8 with export tag matching the exporter) using Lipofectamine 3000 (ThermoFisher) according to manufacturer’s instructions. Media was replaced with 1 mL of fresh media at 8 hours after transfection. At 50 hours after transfection, imaging was performed using a Nikon Ti Eclipse inverted confocal microscope equipped with a 50 μm pinhole spinning disk (Yokagawa), 60x Plan/Apo Ph3 DM oil objective (1.4 numerical aperture), and Andor Zyla 4.2 sCMOS camera. Background subtraction and independent rescaling of each color channel intensity to an identical range across samples was performed using scikit-image (0.19.2)^84^.

#### Morphology of cells stably exporting RNA

To image cells stably expressing RNA exporters, as shown in Figure S4E, we used the cell lines cFH38, which expresses the RNA exporter Gag-MCP and the reporter RNA mCherry-MS2×8, and cFH16, which expresses only the reporter RNA mCherry-MS2×8 (see “Cell line construction”). Wells of 24-well glass-bottom plates were coated with Poly-D-Lysine (ThermoFisher), then cFH38 or cFH16 cells were plated with 50,000 cells per well in media containing 1 ng/μL doxycycline hydrochloride to induce RNA exporter expression (Sigma). At 71 hours after plating, imaging was performed using a Nikon Ti Eclipse inverted confocal microscope equipped with a 50 μm pinhole spinning disk (Yokagawa), 60x Plan/Apo Ph3 DM oil objective (1.4 numerical aperture), and Andor Zyla 4.2 sCMOS camera. Background subtraction and independent rescaling of each color channel intensity to an identical range across samples was performed using scikit-image (0.19.2)^84^.

#### Doubling time of cells expressing exporters

For determining the doubling time of cells expressing RNA exporters, as shown in Figure S4B, HEK293T cells were plated on 96-well plates with 8,000 cells per well. Cells were co-transfected the following day with 100 ng of RNA exporter plasmid (30 ng in the case of EPN24-MCP and EPN11-MCP) using Lipofectamine 3000 (ThermoFisher) according to manufacturer’s instructions. At 24 and 48 hours after transfection, media was removed, cells were lifted from the plate by adding 50 μL of Trypsin-EDTA (ThermoFisher) and incubating at 37 C for 5 minutes, and cells were resuspended by adding 50 μL of media. Microscopy confirmed that no cells remained attached to the plate. Cell counts were determined using the Countess 3 automated cell counter (ThermoFisher). Doubling time T_d_ was calculated using the equation T_d_ = Δt / log2(N_2_/N_1_), where Δt is the time difference between the timepoints (24 hours), N_1_ is the cell count at 24 hours, and N_2_ is the cell count at 48 hours. Three replicate wells were counted for each sample at each timepoint and their variance was propagated to the estimate of T_d_. Cells remained sub-confluent at 48 hours after transfection and exhibited stable expression of transfected plasmids based on fluorescent protein markers at both 24 and 48 hour timepoints.

#### Flow cytometry analysis of cellular toxicity

For flow cytometry analysis of cellular toxicity of RNA exporter expression via transfection of expression plasmids, as shown in Figure S4C, HEK293T cells were plated on 24-well plates with 168,000 cells per well. Cells were co-transfected the following day with 500 ng of RNA exporter plasmid (150 ng in the case of EPN24-MCP) and 500 ng of mCherry-MS2×8 reporter plasmid using Lipofectamine 3000 (ThermoFisher) according to manufacturer’s instructions. Media was replaced with 1 mL of fresh media at 8 hours after transfection. At 71 hours after transfection, media was removed, cells were lifted from the plate by adding 200 μL of Trypsin-EDTA (ThermoFisher) and incubating at 37 C for 10 minutes, and cells were resuspended by adding 160 μL of media.

For flow cytometry analysis of cellular toxicity of RNA exporter expression from stable genomically integrated transgenes, as shown in Figure S4F, cFH38 or cFH16 cells were plated with 40,000 cells per well in a 48-well plate in media containing 1 ng/μL doxycycline hydrochloride to induce RNA exporter expression (Sigma). Media was changed after 24 hours. At 48 hours after plating, media media was removed, cells were lifted from the plate by adding 200 μL of Trypsin-EDTA (ThermoFisher) and incubating at 37 C for 10 minutes, and cells were resuspended by adding 160 μL of media.

In both cases, cells were stained using DRAQ7 (ThermoFisher) at 3 μM for 10 minutes on ice. Cells were passed through a 35 μm filter and analyzed by flow cytometry on a CytoFLEX S instrument (Beckman Coulter). Data were analyzed using FlowJo software (10.8.1). Because dead cells could exhibit different forward and side scattering characteristics from live cells, we avoided potential undercounting of dead cells by analyzing DRAQ7+ events among all events without gating on forward or side scatter. As a positive control for detection of toxicity, cells were heat killed by incubation at 65 C for 5 minutes, then placed on ice for 5 minutes before staining.

#### Relative abundance of secreted particles

To determine the relative abundances of secreted particles with different exporter and cargo configurations, HEK293T cells were plated in 48-well plates and transfected with 75 ng of EPN24-MCP-T2A-GFP plasmid and 250 ng of mCherry reporter plasmid (with either 12, 4, or no MS2 export tags). As a control, the reporter plasmid was omitted. Media was replaced with 1 mL of fresh media at 8 hours after transfection. Media was harvested at 48 hours after transfection, clarified by centrifugation at 3000 g for 5 minutes, passed through a cellulose acetate filter with 0.45 μm pore size (VWR), and stored on ice at 4 C for 12 hours. 20 μL of media was deposited in a well of a 24-well glass-bottom plate (MatTek), dried by incubation at 37 C, rehydrated with 50 μL of DPBS. Imaging was performed using a Nikon Ti Eclipse inverted confocal microscope equipped with a 50 μm pinhole spinning disk (Yokagawa), 60x Plan/Apo Ph3 DM oil objective (1.4 numerical aperture), and Andor Zyla 4.2 sCMOS camera. To count particles, images were passed through a white tophat filter with a disk-shaped footprint with a radius of 5 pixels using scikit-image (0.19.3)^84^, then spots were detected by applying a Laplacian of Gaussian filter followed by local maximum spot detection, as implemented in the detect_spots() function of Big-FISH (0.6.2)^85^.

The relative abundance of particles across conditions was normalized using the particle count of media from cells transfected with the EPN24-MCP and mCherry-MS2×12 expression plasmids as the reference, as shown in Figure S2D. These experiments based on imaging of fluorescent protein tags revealed that the number of particles present in cell culture supernatant was, within ~2-fold, independent of the number of tandem repeats of the MS2 tag in the cargo RNA, as well as the presence or absence of cargo RNA (Figure S2D). Taken together with the positive correlation between total exported RNA and the number of MS2 tags (Figure S2B), this result indicates that the number of RNA molecules encapsulated per particle increased with the number of MS2 tags, saturating at ~8 tags. However, this approach was not suitable for determining the absolute mean number of RNA molecules per particle due to potential undercounting of particles.

#### Cell line construction

Stable cell lines used in this study are listed in the Key Resources Table. To create a stable monoclonal doxycycline-inducible RNA exporter cell line (denoted cFH14.1), we used the PiggyBac transposon system (System Biosciences). HEK293 cells were transfected in a 24-well plate with Gag-MCP-T2A-GFP in a PiggyBac expression backbone containing a blasticidin-selectable marker, transferred to a 12-well plate after 24 hours, and selected with 12.5 ng/μL blasticidin (Invivogen). Selected cells were induced with 1 ng/μL doxycycline hydrochloride (Sigma) for 4 days, then single GFP+ cells were sorted into individual wells of a 96-well plate. Cells were allowed to recover in growth media for 12 days, then supplemented with 12.5 ng/μL blasticidin as they expanded to generate cell stocks.

To create stable drug-resistant cell lines, as shown in Figure 5, we genomically integrated transgenes conferring resistance to puromycin (denoted cFH29) or zeocin (denoted cFH30) into cFH14.1 cells. Cells were plated in a 24-well plate and transduced with lentivirus expressing a puromycin or zeocin resistance gene under control of the EF1 promoter at multiplicity of infection (MOI) of 0.1, transferred to a 12-well plate, and selected with 1 ng/μL puromycin dihydrochloride (Gibco) or 400 ng/μL zeocin (ThermoFisher). Selected cells were expanded and stored in liquid nitrogen before being thawed for further experiments.

To create stable reporter cell lines, as shown in Figure 2, we genomically integrated transgenes consisting of mCherry with (denoted cFH16) or without (denoted cFH15) MS2 export tags under control of the EF1 promoter into HEK293T cells. Cells were plated in a 6-well plate and transduced with lentivirus expressing either mCherry or mCherry-MS2×8 at multiplicity of infection (MOI) of 0.05. Cells were sorted 4 days later to select for mCherry-expressing cells, using gates at a similar expression level in both cell lines (with or without export tag). Cells were expanded and stored in liquid nitrogen before being thawed for further experiments.

To create stable cell lines expressing the Gag-MCP exporter and mCherry-MS2×8 reporter RNA (denoted cFH38), as shown in Figure S1I, we genomically integrated transgenes consisting of mCherry with MS2 export tags (MS2×8) under control of the EF1 promoter into cFH14.1 cells. Cells were plated in a 6-well plate and transduced with lentivirus expressing mCherry-MS2×8 at multiplicity of infection (MOI) of 0.05. Cells were sorted 6 days later to select for mCherry-expressing cells. Cells were expanded and stored in liquid nitrogen before being thawed for further experiments.

#### Monitoring population dynamics

##### Viral barcode library design and production

To genetically label clones with exportable RNA barcodes, we created diverse lentiviral barcode libraries. We cloned a gene fragment consisting of the EF1 promoter, an mCherry fluorescent protein marker, eight tandem repeats of the MS2 stem loop aptamer (denoted MS2×8), woodchuck hepatitis virus post-transcriptional regulatory element (WPRE), and a barcode cloning region (containing restriction enzyme digestion sites used for the cloning procedure described below) into the pCDH lentivirus backbone vector (System Biosciences). After cloning to insert the diverse barcode, the barcode region contained a 5 bp viral index barcode, which is shared by members of a barcode library (specifically, viral index 1 or 2), and a 27 bp clone barcode, which consists of random bases alternating between A/T and G/C to ensure balanced GC-content (denoted WSWSWSWSWSWSWSWSWSWSWSWSWSW, where W indicates A or T, and S indicates G or C).

To clone the diverse barcode library, we first generated a diverse pool of barcode inserts. We synthesized a 60 bp DNA oligo containing hand-mixed random bases and flanking primer binding and restriction digestion sites (IDT) (denoted oFH181 or oFH182 for viral index 1 or 2, respectively). We performed primer extension using this DNA fragment and a complementary primer (oFH180), each at 10 uM, with KAPA Hifi Ready Mix (Roche) and the following thermal profile: 95 C for 3 minutes, 98 C for 20 seconds, 50 C for 15 seconds, and 72 C for 1 minute. We purified the products using the DNA Clean and Concentrate-5 kit (Zymo), digested them using KpnI and NotI (ThermoFisher), and purified the products again using the DNA Clean and Concentrate-5 kit (Zymo). We digested the vector using KpnI and NotI (ThermoFisher), dephosphorylated it using shrimp alkaline phosphatase (ThermoFisher), and purified it using the DNA Clean and Concentrate-5 kit (Zymo). We ligated the barcode insert and vector at a molar ratio of 10:1 (insert:vector) using Ligation Mighty Mix (Takara) by incubating at 16 C for 12 hours, then purified the products using the DNA Clean and Concentrate-5 kit (Zymo). To remove residual uncut vector backbone, we digested this product using SmiI (ThermoFisher), purified the products using the DNA Clean and Concentrate-5 kit (Zymo), and eluted in nuclease-free water. We transformed this DNA into Endura cells (Lucigen) by electroporation using the Gene Pulser Xcell system (Biorad) with a 1 mm cuvette at 10 uF, 600 ohms, and 1800 V following manufacturer’s instructions. We recovered the transformation products by adding 975 μL of recovery media, incubated at 37 C rotating at 250 rpm for 1 hour, plated the liquid on LB Lennox agar (Sigma) bioassay plates, and incubated the plates at 30 C for 16 hours. We scraped all colonies off the plates and extracted plasmid DNA using the ZymoPure II Maxiprep kit (Zymo) according to manufacturer’s instructions, except with elution buffer prewarmed to 50 C and elution performed on column for 10 minutes and including EndoZero treatment. The final plasmid sequence was verified using nanopore sequencing (Primordium).

To prepare lentivirus, HEK293T cells were plated on 10 cm dishes with 6,000,000 cells per dish. Cells were co-transfected the following day with 10 ug of lentiviral transfer plasmid, 10 ug of psPAX2, and 10 ug of pMD2.G packaging plasmids using calcium phosphate. Media was harvested at 48 hours after transfection and stored at −80 C until further use.

##### Barcode library diversity

To characterize the diversity of the barcode libraries, as shown in Figure S5A, Figure S5B, and Figure S5C, we performed deep sequencing of the barcode region. Plasmid was digested using KpnI and MauBI (sites that flank the barcode region) (ThermoFisher) and Ampure XP beads (Beckman Coulter) were used to perform double-sided size selection for the 107 bp product. Sequencing libraries were prepared using the KAPA HyperPrep kit (Roche) following manufacturer’s instructions using 120 ng of input DNA and an adapter molar ratio of 100:1 (adapter:insert). This PCR-free library preparation method does not introduce amplification noise that would distort barcode abundances. Product was subjected to double-sided size selection using Ampure XP beads (Beckman Coulter) at bead ratios of 0.5X followed by 2X to purify the target band at ~350 bp and remove residual undigested plasmid. Libraries were sequenced using the Illumina MiSeq platform with 5M paired-end 300 bp reads per sample.

To identify barcode sequences within each read, we found the sequences that flank the barcode region (GCGGCGCGCC and GCGGCCGCAA). We extracted the barcode sequence as the sequence located between those flanking sequences. Most reads (>90%) matched the expected barcode length of 32 bp and the remainder were predominantly accounted for by uncut vector backbone lacking a barcode insert. Total barcode diversity was estimated using the Chao1 capture-recapture estimator^78^ based on barcodes observed in replicate resamplings at varying depths, as shown in Figure S5A. Collision probability (defined as the fraction of cells at start of experiment which share a barcode due to coincidence of independent barcoding events, rather than common clonal origin) was estimated by resampling as follows. For a given number of cells N, we sampled N barcodes without replacement from the observed barcode pool (with sampling probability proportional to the barcode’s abundance). We calculated the fraction of the sampled barcodes that were unique within the sample, designated p, then the collision probability was 1 - p. This probability is shown in Figure S5C as a function of the number of cells N.

##### Cell culture for population dynamics

For monitoring population dynamics, as shown in Figure 5 and schematized in Figure 5C, we prepared genetically barcoded drug-resistant populations and longitudinally collected supernatant containing exported RNA barcodes. Puromycin- and zeocin-resistant populations of cFH14.1 were prepared as described above and plated on 6-well plates with 400,000 cells/well in media containing 1 ng/μL doxycycline hydrochloride to induce RNA exporter expression (Sigma). Cells were transduced with lentiviral barcode libraries at multiplicity of infection (MOI) of 0.05 with 8 ng/μL polybrene (Millipore Sigma). Two different lentiviral barcode libraries having distinct 5 bp viral index barcodes (viral index 1 and viral index 2) were used to label the two different drug-resistant populations. At 48 hours after transduction, cells were washed with Trypsin-EDTA 0.05%, lifted, and resuspended for cell sorting. mCherry+ GFP+ cells were purified by flow cytometry using a Sony MA900 flow cytometer with 100 μm chip size in targeted mode, sort mode “purity”, and sample pressure of 7. mCherry and GFP indicate expression of the barcoded reporter transcript and the RNA exporter Gag-MCP, respectively. 5,000 cells of each drug-resistant population (puromycin or zeocin-resistant) were sorted into a single well of a 96-well plate containing 400 μL media and 1 ng/μL doxycycline hydrochloride (Sigma). Selection drug, either 1 ng/μL puromycin dihydrochloride (Gibco) or 400 ng/μL zeocin (Invitrogen), was added to each well. Media was collected daily at timepoints spaced 24 hours apart and replaced with 400 μL of fresh media containing doxycycline and selection drug where appropriate. Media was clarified by centrifugation at 3000 g for 5 minutes, passed through a cellulose acetate filter with 0.45 μm pore size (VWR), and stored at −80 C until processing. The first timepoint was collected 24 hours after sorting was complete.

##### Synthetic RNA standard

We used a synthetic RNA spike-in standard to normalize clone barcode abundance across samples. This standard RNA was identical to the exported clone barcode RNA, including having identical GC content, except that it contained a fixed 32 bp barcode sequence in place of the random sequence. RNA was synthesized by *in vitro* transcription as described above.

##### Sequencing library preparation

We devised a simple PCR-based library preparation method for reading out exported RNA barcode abundances from supernatant, as shown in Figure S5D. We diluted the synthetic spike-in RNA standard to 170 aM and added 4 μL of this solution, equivalent to 410 molecules of standard RNA, to 560 μL of Buffer AVL (Qiagen). RNA was then extracted from 140 μL of collected media using the Viral RNA Mini kit (Qiagen) according to manufacturer’s instructions with inclusion of carrier RNA and eluted in 42 μL of Buffer AVE (Qiagen). Reverse transcription was performed using SuperScript IV Reverse Transcriptase (ThermoFisher) according to manufacturer’s instructions with 10 μL of template RNA (1 – 1.5 ug), 100 nM target-specific primer (oFH99), 500 μM dNTPs, 5 mM dithiothreitol, and 20 units of SUPERase-In RNase inhibitor (ThermoFisher). The entire 20 μL product was used as input for PCR with KAPA Hifi Ready Mix (Roche) with 300 nM forward and reverse primers containing Illumina adapters and the following thermal cycling profile: 95 C for 3 minutes, 35 cycles of 98 C for 20 seconds, 67 C for 15 seconds, and 72 C for 15 seconds, and final extension of 72 C for 1 minute. These PCR primers are denoted oFH124, oFH125, oFH135–144 (forward), oFH126, oFH127, or oFH145–154 (reverse). Product was purified using Ampure XP beads (Beckman Coulter) at 1.5X bead ratio, purified again using Ampure XP beads at 1X bead ratio, and eluted in 35 μL of 10 mM Tris-Cl pH 8.5. Libraries were sequenced on the MiSeq platform (Illumina) with >400,000 paired-end 75 bp reads per sample (mean of ~1M reads per sample).

For characterizing the accuracy of barcode abundances measured in exported RNA, as shown in Figure 5D, we collected cells at the final timepoint of the experiment by adding 350 μL ice-cold Buffer RLT containing 2-mercaptoethanol directly to the well and scraping with a pipette tip. RNA was extracted using the RNeasy Mini kit (Qiagen). Barcode amplicon libraries were prepared and sequenced alongside supernatant samples, as described above except using 5 μL of RNA (250 – 1000 ng) as template for reverse transcription. For characterizing the reproducibility of the measurement, as shown in Figure 5E, RNA was extracted from an additional replicate 140 μL aliquot of supernatant, and libraries were prepared and sequenced alongside the other samples.

##### Sequence preprocessing

To identify and count clone barcode sequences, paired-end reads were merged using FLASH (1.2.11)^86^ with parameters “--maximum-overlap=75 --max-mismatch-density=0.5”. Barcodes were extracted from the merged reads as the sequence located between the barcode-flanking sequences (5’-GCGGCCGC and 5’-GGCGCGCC), confirmed to match the designed 32 bp sequence (NNNNNWSWSWSWSWSWSWSWSWSWSWSWSWSW), and separated into the 5 bp viral index barcode and the 27 bp clone barcode. Viral indexes were assigned to references, requiring perfect identity to a reference sequence (discarding reads lacking perfect identity). Within each population marked by a distinct viral index, clone barcodes were clustered to correct PCR and sequencing errors at a Levenshtein distance of one using Starcode (1.4)^87^ with parameters “--dist 1 --threads 16”.

We developed an algorithm to distinguish clone barcodes from erroneous sequences based on the distribution of read counts. Erroneous sequences can arise from residual PCR and sequencing errors that were not corrected by Starcode, resulting in sequences that are represented by few reads (in >99% of cases, only one read). To remove these sequences in an unbiased manner, we used a knee point filter similar to that used in 10X Genomics CellRanger 2.2 (ref. ^88^). The knee point threshold was set to 1% of the 99th percentile of read abundance among the top N = 5,000 barcodes, where N is the expected maximum number of barcodes (here N = 5,000 because 5,000 cells per population were sorted into the well to initiate the experiment). Barcodes were further filtered to exclude those with fewer than 10 reads. To enhance overall detection sensitivity for clone barcodes having changing abundance (e.g. barcodes that went extinct), filtering was performed separately for each timepoint, then resulting calls for each barcode were propagated across timepoints, such that a barcode was excluded if and only if the filter excluded it at all timepoints (equivalently, a barcode was included as genuine if the filter included it at any timepoint).

Barcode abundances were normalized using the spike-in standard. More specifically, the total read count of the standard was determined based on a perfect match with its 5 bp reference population barcode. We then rescaled the read count of each clone barcode to counts per million of standard (CPMS) (by multiplying the read count of the barcode by 1,000,000 and dividing by the total read count of standard within the same library). Finally, a normalized barcode abundance matrix was formed with each clone barcode represented by a row and each timepoint represented by a column.

##### Analysis of clonal population dynamics

Total abundance of each drug-resistant or -sensitive population was determined by summing the abundance of all clones belonging to that population (in CPMS space) and plotted at each timepoint, as shown in Figure 5F and Figure S6C. To display relative clone abundance, as shown in Figure 5H and Figure S6C, we randomly sampled 100 clone barcodes from each population, normalized their abundance to sum to 1, and plotted these normalized abundances at each timepoint.

To determine clone growth rates, we fit an exponential growth model *f(t)* = *Ae*^*kt*^ to each clone abundance trajectory, where *f(t)* is clone abundance (in units of CPMS) and *t* is time (days), using non-linear least squares with initial parameter guesses of *A* = 10,000 and *k* = 0, as implemented in the curve_fit() function of scipy (1.4.1). To conservatively estimate growth rates, f(t) was set equal to the detection limit in samples in which a clone was not detected, thus providing a detection-limited estimate of the growth rate. Examples of fits are shown in Figure S6B. We excluded fits with goodness of fit (R^2^) < 0.9, which predominantly removed clones that were detected at only one or two timepoints. Distributions of fitted growth rates *k* are shown in Figure 5I and Figure S6D.

##### Rarefaction analysis

Rarefaction analysis was performed to ensure that sequencing depth was sufficient to saturate clone discovery. We sampled 1, 2, 5, 10, 20, 50, 100, 200, 500, 1,000, 2,000, 5,000, 10,000, 20,000, 50,000, 100,000, 200,000, 500,000, 1,000,000, 2,000,000, 5,000,000, or the totality of reads per library with replacement. The number of unique barcode sequences was determined and plotted as a function of the number of reads sampled, as shown in Figure S5H or Figure S6A.

##### Reproducibility of population dynamics reporter

For characterizing the reproducibility of the reporter system, as shown in Figure 5E, libraries were prepared from two distinct aliquots of supernatant, as described above, and their reads were preprocessed separately. The fraction of barcodes detected in both replicates was determined. The correlation between the abundances of all barcodes (including barcodes that were only detected in one of the replicates) was calculated.

##### Accuracy of population dynamics reporter

For characterizing the accuracy of the reporter system, as shown in Figure 5D, we used clone barcodes detected in cellular RNA as a “ground truth” reference set. We compared the abundances of barcodes detected in exported RNA at the final timepoint of the experiment against this reference set. To construct the reference set, sequencing reads obtained from cellular RNA were preprocessed as described above through barcode clustering using Starcode. Clone barcode detection by knee point threshold was performed on barcodes in cellular RNA. Detection sensitivity in exported RNA was evaluated against this set by determining the fraction of barcodes detected in cellular RNA that were also detected in exported RNA. Conversely, nearly all barcodes detected in exported RNA were also detected in cellular RNA (46,132 out of 46,134 or 99.996%), confirming the high sensitivity of detection in cellular RNA and validating its use as a reference. Finally, clone abundance in cellular and exported RNA were plotted against each other and their correlation was calculated (including barcodes that were detected exclusively in one dataset).

##### Sensitivity of population dynamics reporter

To determine the sensitivity of detecting exported RNA originating from single cells, we prepared, barcoded, and sorted cell populations as described above, except we sorted only 10 barcode-labeled zeocin-resistant cells into a single well of a 48-well plate (with eight replicate wells) together with a carrier population of 29,990 HEK293T cells. Media was harvested 24 hours after sorting. We confirmed by microscopy that most labeled cells remained solitary at this time (data not shown), suggesting that they had not divided and represented single-cell clones, as expected based on the ~24-hour doubling time of HEK293 cells. Sequencing libraries were prepared from exported RNA as described above. Reads were preprocessed as described above through the step of barcode clustering using Starcode. Then clone barcode detection by knee point threshold was performed as described above, except using N = 10 (because a maximum of N = 10 cells are expected based on cell sorting). The results of this clone barcode detection procedure are shown in Figure S5G and reveal the number of clone barcodes detected in an unbiased manner based on the read count distribution.

To determine the rate at which cells survived single-cell sorting, we sorted one HEK293T cell per well of three 96-well plates, with sorting performed as described above. We cultured the cells for 7 days, then counted the number of wells having surviving cells by microscopy. Out of 288 total wells, 185 wells contained surviving cells, providing an estimated survival rate of 64 ± 6% (mean ± 95% binomial confidence interval based on the asymptotic normal approximation).

Because cell survival rates can depend on the number of cells sorted into a well, which could in turn affect estimates of reporter sensitivity, estimates of cell survival were refined by analyzing results from bulk sorting of 10,000 barcoded cells per well. More specifically, in the population dynamics experiments performed in the absence of drug selection, we sorted 10,000 cells together into each well, then recovered 3,956 ± 264 (mean ± s.d. of 2 experiments) clone barcodes from each population after 6 days of culture, corresponding to a survival rate of 40 ± 3%. Thus, the cell survival rate of 64 ± 6% estimated from single-cell sorting does not underestimate the survival rates obtained when sorting larger populations.

These results also demonstrate sensitive detection of exported reporter transcripts with both exporter and reporter genes stably integrated in the genome, and the reporter gene present at single copy.

##### RNA exporter silencing dynamics

To characterize RNA exporter expression dynamics during the population dynamics experiment, as shown in Figure S5E, monoclonal inducible RNA exporter-expressing cells (cFH14.1) were plated on a 6-well plate, induced with doxycycline hydrochloride (Sigma) at 1 ng/μL for 2 days, and purified GFP+ cell populations were sorted into wells of a 96-well plate. For the zero timepoint, the sorted cells were immediately analyzed. For subsequent timepoints at days 1, 3, 5, and 7, the cells were lifted from the plate using 50 μL of Trypsin-EDTA (ThermoFisher), resuspended by addition of 150 μL of media, passed through a 35 μm filter, and analyzed on a CytoFLEX S flow cytometer (Beckman Coulter). Data were analyzed by using FlowJo software (10.8.1) to gate for live single cells based on forward and side scatter, then calculating the fraction of cells expressing GFP at each timepoint.

#### Delivery of Cre recombinase mRNA

To test delivery of Cre-encoding mRNA by EPN24-MCP or Gag-MCP, as shown in Figure 6 and Figure S7B, we exported RNA from sender cells and transferred their conditioned media to receiver cells, which activate RFP expression upon Cre recombination. HEK293T cells were plated on 12-well plates with 300,000 cells per well. Cells were co-transfected the following day with 375 ng of RNA exporter plasmid, 1250 ng of Cre cargo plasmid, and 50 ng of VSV-G fusogen plasmid using Lipofectamine 3000 (ThermoFisher) according to manufacturer’s instructions. Media was replaced with 1 mL of fresh media at 8 hours after transfection. The following day, HEK293 Color-Switch loxP/GFP/RFP (hereafter, Cre reporter) cells (Creative Biogene) were plated on 24-well plates with 50,000 cells per well. At 48 hours after transfection, media was harvested, clarified by centrifugation at 3000 g for 5 minutes, and passed through a cellulose acetate filter with 0.45 μm pore size (VWR). Media was removed from the Cre reporter cells and replaced with the conditioned media. Reporter cells were incubated for 72 hours, lifted from the plate, passed through a 35 μm filter, and analyzed on a CytoFLEX S flow cytometer (Beckman Coulter). Data were analyzed by using FlowJo software (10.8.1) to gate for live single cells based on forward and side scatter then for reporter cassette-expressing GFP+ cells, and calculating the fraction of such cells expressing RFP.

Effects of fusogen plasmid dosage, as shown in Figure S7C, were characterized in similar experiments, except varying the amount of VSV-G fusogen plasmid. Effects of RNA export enhancers on delivery, as shown in Figure S7D, were characterized using similar experiments except with co-transfection of export enhancer plasmids (1250 ng each) together with other system components.

#### Delivery and expression of mCherry mRNA

To measure the dynamics of delivery and expression of mCherry fluorescent protein by the EPN24-MCP system, HEK293T cells were plated on 12-well plates with 300,000 cells per well. Cells were co-transfected the following day with 375 ng of EPN24-MCP exporter plasmid, 50 ng of VSV-G fusogen plasmid, 500 ng of mCherry-MS2×8 cargo plasmid, and 625 ng each of CIT and NEDD4LΔC2 export enhancer plasmids. Exporter plasmid was omitted as a control. Media was replaced with 1 mL of fresh media at 8 hours after transfection. At 24 hours after transfection, HEK293 cells were plated on 24-well plates with 50,000 cells per well to serve as receiver cells. At 48 hours after transfection, the conditioned media was harvested from sender cells, clarified by centrifugation at 3000 g for 5 minutes, and passed through a cellulose acetate filter with 0.45 μm pore size (VWR). Media was then removed from the receiver cells and replaced with 1 mL of conditioned media. Receiver cells were incubated for 0, 3, 6, 12, 24, 32, or 48 hours after media transfer, lifted from the plate, passed through a 35 μm filter, and analyzed on a CytoFLEX S flow cytometer (Beckman Coulter). Data were analyzed by using FlowJo software (10.8.1) to gate for live single cells based on forward and side scatter, then for mCherry-expressing cells, and fraction of cells expressing RFP was calculated.

#### Delivery of two fluorescent protein mRNAs

Delivery of two cargo RNAs, as shown in Figure 6E, was tested in similar experiments, except with co-transfection of cargo plasmids for both mCherry and BFP at 500 ng and 2000 ng, respectively, as well as 625 ng each of CIT and NEDD4LΔC2 plasmids.

#### Cell-to-cell delivery of mRNA in co-culture

To test delivery of mRNA in a co-culture context, as shown in Figure 7, HEK293T cells were plated on 12-well plates with 300,000 cells per well. Cells were co-transfected the following day with 375 ng of EPN24-MCP exporter plasmid, 50 ng of VSV-G fusogen plasmid, 1250 ng of Cre-MS2×12 cargo plasmid, 625 ng each of CIT and NEDD4LΔC2 export enhancer plasmids, and 200 ng of TagBFP marker plasmid. Exporter plasmid or export and fusogen plasmids were omitted as controls. BFP was used as a marker for the sender cell population. At 8 hours after transfection, these cells were lifted and 800,000 cells were plated into the periphery of a 6-well plate using 4-chamber cell culture inserts (Ibidi). 10,000 HEK293 Cre reporter cells were plated into each of the four chambers in the center of the insert. Inserts were removed 12 hours later using forceps and cell culture media was replaced with fresh media supplemented with 8 μg/mL polybrene (Sigma). Cells were cultured with media changes as needed to maintain cell health every 24 to 48 hours. At 7 days after transfection, imaging was performed using an EVOS FL Auto imaging system (ThermoFisher) equipped with a 10x objective and the TagBFP (390/18 nm excitation; 447/60 nm emission) and Texas Red filter cubes (585/29 nm excitation; 628/32 nm emission) using the scan function of the EVOS software. Stitched image files were downscaled by 10-fold in each dimension and independent rescaling of each color channel intensity to an identical range across samples was performed using scikit-image (0.19.2)^84^. Mean pixel intensity in the RFP channel was calculated in circular shells of varying radii, as shown in Figure 7C.

### Quantification and statistical analysis

Quantification and statistical analysis was performed using Python version 3.7.7. Details of statistical tests, including statistical tests used, exact number of samples, and dispersion and precision measures, are indicated in the appropriate figure legend.

### Additional resources

Detailed protocols and troubleshooting information for RNA export experiments are available at http://rnaexport.org.

## Supplementary Material

1 **Figure S1. Design and characterization of engineered viral RNA exporters, Related to** Figure 1. (A) Diameter of virus-like particles (VLPs) secreted by cells expressing viral RNA exporters was measured using dynamic light scattering (DLS) after purification by ultracentrifugation through a 20% sucrose cushion. Labels indicate mean diameter. Note that particle diameters measured by DLS are larger than those measured by electron microscopy because aggregates cannot readily be distinguished from single particles by DLS. (B) Negative-stain transmission electron microscopy revealed that the supernatant of HEK293T cells transfected with expression plasmid of mCherry RNA alone (without an RNA exporter) lacked particles with >50 nm diameter. (C) Loss of RNA due to the cleanup steps of clarification (by centrifugation) and filtration was quantified using RT-qPCR. (D) To confirm that the RT-qPCR assay faithfully measures RNA abundance, rather than potential contaminants such as DNA plasmids, we omitted reverse transcription (RT) prior to qPCR. Omitting RT substantially reduced the apparent number of RNA molecules detected for all samples, indicating that the background signal from DNA contamination is lower than the foreground signal originating from RNA (cDNA after reverse transcription). Each dot represents one technical replicate; colors represent biological replicates (independent cell culture wells); bar indicates the mean of replicates. Consistency across biological replicates confirms the reproducibility of the assay. (E) Rate of RNA export was determined based on accumulation of RNA in culture supernatant after transfection using linear regression. Data for (E) are mean and standard deviation of three biological replicates. (F) Rate of RNA export can be tuned by varying the number of MS2 export tag repeats in 3’ UTR of the cargo RNA. (G) Rates of RNA export can also be tuned by the expression level of the exporter Gag-MCP and the cargo RNA, as shown by transfection of varying amounts of exporter or reporter expression plasmids. (I) RNA was successfully exported by expression of the Gag-MCP exporter and cargo RNA from genomically integrated transgenes. (J) Designs of viral RNA exporters, in which viral capsid proteins are fused to an RNA binding domain. Labels refer to domains of MMLV or HIV Gag, except MCP, which denotes MS2 bacteriophage coat protein, and Zip, which denotes GCN4 leucine zipper. Subscript indicates the domain upstream of fusion (e.g. MCP_Zip_ indicates that MCP is fused to the C-terminus of Zip). The “∆pol” variant lacks a slippery frameshift-inducing sequence within the HIV Gag coding sequence. (K) Engineered viral RNA exporters secreted target RNA bearing export tags into culture supernatant with varying degrees of efficiency and specificity. Wild-type HIV Gag was not evaluated for RNA export because the HIV packaging signal sequence has not been unambiguously defined. MSx12 indicates twelve MS2 hairpins in a tandem array. (L) Expression levels of cargo RNA molecules cannot account for differences in RNA export efficiency. (M and N) Stability of RNA packaged and secreted by Gag-MCP or *in vitro* transcribed mRNA (not packaged by an RNA exporter) during incubation in cell culture supernatant (M) or whole mouse blood (N) at 37 C. Data for (M) and (N) are mean and standard deviation of 3 technical replicates. In (C), (F), (G), (H), (I), (K), and (L), each dot represents one technical replicate. In (C), (F), (G), (H), and (I), line indicates the mean of replicates. In (K) and (L), bar indicates the mean of replicates. In (D), (E), (G), (H), (I), and (K), dashed line indicates lower limit of quantification.
**Figure S2. Design and characterization of protein nanocage-based RNA exporters, Related to** Figure 2. (A) The amount of RNA exported by EPN24-MCP and Gag-MCP was similar across different durations of accumulation, indicating that the rate of RNA export by EPN24-MCP is similar to that of Gag-MCP. Note that measurements at 48 or 72 hours were performed in separate experiments, precluding direct comparison across timepoints. (B) Rate of RNA export can be tuned by varying the number of MS2 export tag repeats in 3’ UTR of the cargo RNA. (C) Diameter of particles secreted by cells expressing nanocage-based RNA exporters was measured using dynamic light scattering after purification by ultracentrifugation through a 20% sucrose cushion. Labels indicate mean diameter. (D) Counting particles by imaging a green fluorescent protein tag revealed that the relative number of secreted EPN24-MCP particles was, within ~2-fold, independent of the number of export tags in cargo RNA and its presence or absence. (E and F) Stability of RNA packaged and secreted by EPN24-MCP or *in vitro* transcribed mRNA (not packaged by an RNA exporter) during incubation in cell culture supernatant (E) or whole mouse blood (F) at 37 C. Data for (D), (E), (F) are mean and standard deviation of 3 technical replicates. (G, H, and I) To evaluate the cargo capacity of the EPN24-MCP system, we measured the expression level in exporting cells (G) and abundance in supernatant (H) of cargo RNAs of increasing length. (I) Export efficiency was, within 3-fold, insensitive to cargo RNA length up to 9.8 kb, as revealed by computing the ratios of RNA abundances in supernatant and cells, which is a metric of efficiency that accounts for the availability of cargo RNA. Data for (G), (H), and (I) are mean and standard deviation of 3 biological replicates. (J) To test enhancement of RNA export by modulators of cellular secretion pathways, we co-expressed the EPN24-MCP RNA export system with a panel of 6 candidate modulators. (K) Two activators of the ESCRT pathway, NEDD4L and CIT, enhanced RNA export rates. p-values were calculated using Student’s two-sided t-test. (L) Co-expressing an inhibitor of ESCRT-dependent secretion, VPS-E228Q, reduced RNA export rates. (M) To test modular design of RNA targeting, we fused the sequence-specific RNA binding protein PP7 coat protein (PCP) to EPN24, yielding EPN24-PCP. This system demonstrated efficient export of cognate cargo RNA tagged with the PP7 stem-loop aptamer, albeit with lower specificity than EPN24-MCP. MS2 and PP7 export tags each consisted of 12 tandem repeats of the corresponding stem-loop aptamer (denoted MS2 or PP7). In (A), (B), (K), (L), and (M), each dot represents one technical replicate. In (A), (K), (L), and (M), bar indicates the mean of replicates. In (B), line indicates the mean of replicates and dashed line indicates lower limit of quantification.
**Figure S3. Additional genome-scale characterization of RNA export efficiency and specificity, Related to** Figure 3. (A) Engineering of RNA exporters enhanced their specificity for target RNA and thereby reduced secretion of endogenous (non-target) RNA. Plot shows the abundance of total endogenous RNA in supernatant in the presence of each exporter compared to its absence. (B) The improved specificity of engineered RNA exporters manifested as reduced secretion of endogenous (non-target) RNA spanning the entire transcriptome. Each violin shows the genome-wide distribution of transcript enrichments in supernatant in the presence of the exporter compared to its absence. Successive design generations preserved efficient secretion of target RNA (pink star), but exhibited improved specificity, as manifested by greater enrichment of target RNA compared to other transcripts. (C) Off-target RNA export enhanced the detection rates of endogenous cellular transcripts in cell culture supernatant. Genes were binned according to their expression level in the cellular transcriptome, then the detection rate of genes within each bin was calculated (with detection defined as CPM > 1). CPM denotes counts per million. (D) Expression of MMLV Gag enhanced the abundance of non-target GAPDH mRNA in supernatant, as measured by RT-qPCR, confirming the high off-target export activity of MMLV Gag. Lower enhancement was observed in the presence of its target RNA tagged with the packaging signal Psi, indicating that target and non-target RNA compete for packaging by MMLV Gag. (E) For Gag-MCP, higher export of non-target GAPDH mRNA was observed at lower expression levels of its target RNA, indicating that target and non-target RNA also compete for packaging by Gag-MCP. In (D) and (E), each dot represents one technical replicate. In (D), line indicates mean of the replicates. In (E), bar indicates mean of the replicates. ND denotes not detected.
**Figure S4. RNA exporters are non-toxic and do not perturb cellular morphology, growth, or transcriptome, Related to** Figure 1 and Figure 2. (A) Cells expressing RNA exporters, as indicated by a co-translational green fluorescent protein (GFP) marker, appeared morphologically normal in comparison to cells expressing only the mCherry fluorescent protein. (B) Growth rates of cells transfected with RNA exporter expression plasmids were similar to those of cells not subjected to this treatment. By contrast, sensitive cells treated with the growth-inhibiting drug zeocin exhibited reduced growth rates, validating the assay. Data for (B) are mean and standard deviation of three biological replicates. (C) Analysis of cellular toxicity using a dead cell stain (DRAQ7) and flow cytometry. Cells expressing RNA exporters maintained low rates of cell death, comparable to negative controls lacking RNA exporter expression (labeled “None”). To avoid excluding dead cells, no gating was performed. Each dot represents one biological replicate and the bar indicates the mean of the replicates (n > 9,800 events per replicate). (D) Sequencing of cellular RNA revealed no detectable perturbation to cellular transcriptomes due to RNA exporter expression. Differential expression analysis compared cells transfected with only fluorescent protein expression plasmids (mCherry, bearing the appropriate export tag, and blue fluorescent protein, BFP, lacking an export tag) against cells transfected with these same plasmids plus an RNA exporter expression plasmid. Only the RNA exporter itself showed significant and reproducible differential expression in each case. In the cases of MMLV Gag and GagZip-MCP, a single endogenous gene surpassed the significance threshold of p < 0.05 for differential expression in a single replicate, but no genes surpassed this significance threshold in all replicate samples. Notably, mitochondrial RNA exhibited no significant differences in abundance, suggesting that cell health was maintained. (E) Cells stably expressing the RNA export system – consisting of the exporter Gag-MCP, as indicated by a co-translational green fluorescent protein (GFP) marker, and reporter mCherry mRNA with export tags – from genomically integrated transgenes appeared morphologically normal in comparison to cells stably expressing only mCherry. (F) Analysis of cellular toxicity using a dead cell stain (DRAQ7) and flow cytometry. Cells stably expressing the Gag-MCP export system maintained low rates of cell death, comparable to negative controls lacking transgene expression (labeled “None”). To avoid excluding dead cells, no gating was performed. In (F), each dot indicates one biological replicate and bar indicates the mean of the replicates.
**Figure S5. Barcode libraries, workflow, and performance of RNA-export based population dynamics reporter system, Related to** Figure 5. (A-C) Diversity of the barcode libraries was characterized by deep sequencing of plasmid DNA using amplification-free library preparation. (A) Lower bound of total barcode diversity within each library was estimated based on observed counts using the Chao1 capture-recapture estimator^78^. (B) Barcode abundance distributions were nearly uniform. (C) Labeling capacity of each library was estimated by using simulations to determine the probability of barcode collisions (coincidence) within labeled cell populations of varying sizes. Data for (C) are the mean and standard deviation of 100 replicate simulations (resamplings). Note range of the y-axis scale. (D) Workflow for counting exported RNA barcodes using sequencing. (E) Flow cytometry revealed spontaneous silencing of RNA exporter expression in a subpopulation of cells during the experiment. Data for (E) are mean and standard deviation of 2 biological replicates per time point. (F) To measure reporter sensitivity, as defined by the minimum number of cells of a given clone that can be reliably detected, we sorted 10 uniquely labeled cells into a single well, cultured them for 24 hours to allow their RNA barcodes to be exported, then collected and sequenced the barcodes from culture supernatant. We estimated the sensitivity as the number of unique barcodes detected per well. (G) Barcodes were detected from 5.2 ± 3.2 cells per well (mean ± s.d. of 10 replicate wells) out of a possible maximum of 10 cells. Because only 64 ± 6% (mean ± 95% confidence interval) of cells survived sorting in control experiments, these results suggest a lower bound of 81% on detection sensitivity for RNA barcodes exported by a single cell at daily time resolution. Plots show the read counts for unique barcodes detected in each well. Dashed line indicates the minimum read count cutoff used to discriminate genuine cell detection events from erroneous barcodes, which was determined using an unbiased knee point detection algorithm (Methods). Note that 11 barcodes were detected in well 6, possibly reflecting the sorting of a doublet. (H) Clone discovery was saturated with respect to sequencing depth. Data for (H) are mean and standard deviation of 10 replicate resamplings.
**Figure S6. Additional characterization of monitoring population dynamics using RNA export and sequencing, Related to** Figure 5. (A) Clone discovery was saturated with respect to sequencing depth. (B) To estimate growth rates of individual clones, a model of exponential growth (equation shown) was fitted to the time-varying abundance of each clone. Examples of model fits and parameters (best fit ± s.d.) are shown for three drug-resistant clones and three drug-sensitive clones grown in the presence of puromycin. Distributions of growth rates of all clones are shown in Figure 5I. (C) Clonal population dynamics of cells cultured without drug selection. Top, collective dynamics of two distinctly labeled populations (puromycin-resistant and zeocin-resistant, which bear different lentiviral barcodes). Middle, clones detected within each population. Bottom, population dynamics of individual clones resolved by tracking unique clone barcodes. The fractional abundances of 100 clones randomly sampled from each population are shown, with each distinctly shaded block indicating a different clone. (D) Distributions of growth rates of individual clones grown without drug. Dashed line indicates population-average growth rate determined independently by cell counting.
**Figure S7. Optimizing and characterizing cell-to-cell delivery of RNA, Related to** Figure 6. (A) Cre reporter consistently saturated at <100% activity with 1000 ng of Cre mRNA transfected, despite variation in the precise level of activity at saturation across different experiments. Data for (A) are the mean and standard deviation of 3 biological replicate wells at each dose. This experiment was performed in parallel with that of Figure S7B. (B) Gag-MCP RNA delivery system failed to deliver functional mRNA cargo encoding Cre to reporter cells. Dashed line indicates maximum activity observed with saturating doses of Cre mRNA transfected into reporter cells. (C) Intermediate amounts of fusogen VSV-G expression plasmid were optimal for efficient and specific RNA delivery. 20:1 molar ratio of VSV-G plasmid to EPN24 plasmid, corresponding to 50 ng of fusogen plasmid in this experiment, was used for subsequent experiments. (D) Enhancers of RNA export, specifically CIT and NEDD4L (identified in Figure S2K), also enhanced RNA delivery. Dashed line shows no change (fold-change of 1) compared to without enhancer. p-values were calculated using Welch’s two-sided t-test. In (B) and (D), each dot represents one biological replicate; bar or solid line indicates the mean of replicates. (E and F) To determine the dynamics of cargo mRNA delivery and expression, we monitored mCherry fluorescence in receiver cells over time by flow cytometry. Data for (E) are mean and standard deviation of 3 biological replicates per timepoint. The distribution of mCherry intensities is shown for one replicate at each timepoint in (F).

## Figures and Tables

**Table:** Key resources table

Reagent or resource	Source	Identifier
**Bacterial and virus strains**		
Stable Competent E. coli (High Efficiency)	NEB	C3040H
Endura ElectroCompetent E. coli	Lucigen	60242-1
**Chemicals, peptides, and recombinant proteins**		
Dulbecco’s Modified Eagle Medium	ThermoFisher Scientific	11960-069
Roswell Park Memorial Institute (RPMI) 1640 Medium with GlutaMAX supplement	ThermoFisher Scientific	72400-047
Alpha Minimum Essential Medium	Irvine Scientific	41127
Fetal bovine serum	Avantor	97068-085
Penicillin-Streptomycin-Glutamine	ThermoFisher Scientific	10378016
Sodium pyruvate	ThermoFisher Scientific	11360070
Minimal Essential Medium Non-Essential Amino Acids	ThermoFisher Scientific	11140050
Trypsin-EDTA (0.05%)	ThermoFisher Scientific	25300054
Dulbecco’s Phosphate Buffered Saline containing calcium and magnesium	ThermoFisher Scientific	14040117
Opti-MEM I Reduced Serum Medium	ThermoFisher Scientific	31985070
Poly-D-Lysine	ThermoFisher Scientific	A3890401
2-mercaptoethanol	ThermoFisher Scientific	21985-023
Uranyl formate	Electron Microscopy Sciences	22450
N1-Methylpseudouridine-5’-Triphosphate	TriLink	N-1081
Deoxynucleotide (dNTP) Solution Mix	NEB	N0447L
CleanCap AG	TriLink	N-7113
DNase I (RNase-free)	NEB	M0303L
Lipofectamine 3000	ThermoFisher Scientific	L3000008
RNase A	ThermoFisher Scientific	EN0531
RNase T1	ThermoFisher Scientific	EN0541
SUPERase In RNase inhibitor	ThermoFisher Scientific	AM2694
Triton X-100	Fisher Scientific	AC327371000
External RNA Controls Consortium (ERCC) synthetic spike-in RNA mix	ThermoFisher Scientific	4456740
DRAQ7 dye	ThermoFisher Scientific	D15106
Puromycin Dihydrochloride	ThermoFisher Scientific	A1113803
Blasticidin	InvivoGen	ANT-BL-1
Hygromycin B Gold	InvivoGen	ANT-HG-1
Zeocin	ThermoFisher Scientific	R25001
Doxycycline Hydrochloride	Sigma Aldrich	D3072
Polybrene	Millipore Sigma	TR-1003-G
FastDigest KpnI	ThermoFisher Scientific	FD0524
FastDigest NotI	ThermoFisher Scientific	FD0596
FastDigest SmiI	ThermoFisher Scientific	FD1244
FastDigest MauBI	ThermoFisher Scientific	FD2084
Shrimp Alkaline Phosphatase	ThermoFisher Scientific	78390500UN
LB Broth with agar (Lennox)	Sigma Aldrich	L2897-250G
SuperScript IV Reverse Transcriptase	ThermoFisher Scientific	18090050
**Critical commercial assays**		
MycoStrip	InvivoGen	rep-mysnc-50
QIAamp Viral RNA Mini Kit	Qiagen	52906
RNeasy Mini Kit	Qiagen	74104
TURBO DNA-free Kit	ThermoFisher Scientific	AM1907
iScript Reverse Transcription Supermix	Biorad	1708841BUN
iQ SYBR Green Supermix	Biorad	1708887
Qubit RNA HS Assay Kit	Fisher Scientific	Q32852
RNA 6000 Pico Kit	Agilent	5067-1513
High Sensitivity D1000 ScreenTape	Agilent	5067-5584
High Sensitivity D1000 Reagents	Agilent	5067-5585
DNA Clean & Concentrator-5 Kit	Zymo	D4014
RNA Clean and Concentrator-5 Kit	Zymo	R1014
HiScribe T7 High Yield RNA Synthesis Kit	NEB	E2040S
SMARTer Stranded Total RNA-Seq v2 Pico Input kit	Takara	634411
KAPA HiFi HotStart ReadyMix	Roche	7958935001
DNA Ligation Mighty Mix	Takara	6023
ZymoPure II Maxiprep kit	Zymo	D4203
KAPA HyperPrep kit (PCR-free)	Roche	KK8503
MiSeq Reagent Kit v3 (150-cycle)	Illumina	MS-102-3001
**Deposited data** (The following data will be deposited prior to publication)		
Raw sequencing reads for genome-scale characterization of cellular and exported RNA	This study	PRJNA934101
Raw sequencing reads for characterizing barcode library diversity	This study	PRJNA943427
Raw sequencing reads for monitoring clonal population dynamics	This study	PRJNA943434
Preprocessed data	This study	Accession ID pending
Raw microscopy images	This study	Accession ID pending
**Experimental models: Cell lines**		
HEK293 cell line	ATCC (American Type Culture Collection)	CRL-1573
HEK293T cell line	ATCC	CRL-3216
HEK293FT cell line	ThermoFisher	R70007
K562 cell line	ATCC	CCL-243
Jurkat cell line	ATCC	TIB-152
C3H/10T1/2 cell line	ATCC	CCL-226
CHO-K1 cell line	ATCC	CCL-61
loxP/GFP/RFP Color-Switch Cre reporter HEK293 cell line	Creative Biogene	CSC-RR0082
cFH14.1 (HEK293 cell line with doxycycline-inducible expression of Gag-MCP-T2A-GFP)	This study	N/A
cFH15 (HEK293T cell line with constitutive expression of mCherry)	This study	N/A
cFH16 (HEK293T cell line with constitutive expression of mCherry-MS2×8)	This study	N/A
cFH29 (HEK293 cell line with doxycycline-inducible expression of Gag-MCP-T2A-GFP and constitutive expression of puromycin-resistance gene)	This study	N/A
cFH30 (HEK293 cell line with doxycycline-inducible expression of Gag-MCP-T2A-GFP and constitutive expression of zeocin-resistance gene	This study	N/A
cFH38 (HEK293 cell line with doxycycline-inducible expression of Gag-MCP-T2A-GFP and constitutive expression of mCherry-MS2×8)	This study	N/A
**Other**		
0.45 μm cellulose acetate syringe filter	VWR	28145-481
UVette 220-1600 nm cuvette	Eppendorf	952010051
Whatman UNIFILTER plates 96-well 0.45 μm pore size, cellulose acetate membrane	Millipore Sigma	WHA77002808
Agencourt AMPure XP beads	Beckman Coulter	A63880
24-well glass bottom plates, No. 1.5 coverslip, 10 mm glass diameter, uncoated	MatTek	P24G-1.5-10-F
Whole mouse blood (CD1)	Innovative Research	IGMSCD1WBK2E10ML
4-chamber cell culture inserts	Ibidi	80469
**Oligonucleotides**		
oFH77 mCherry qPCR forward primer	IDT	CCTCAGTTCATGTACGGCTCC
oFH78 mCherry qPCR reverse primer	IDT	TCGAAGTTCATCACGCGCTC
oFH189 Cre qPCR forward primer	IDT	ACAACTACCTGTTCTGCCG
oFH190 Cre qPCR reverse primer	IDT	GCCTCAAAGATCCCTTCCAG
oFH57 GAPDH qPCR forward primer	IDT	GTCTCCTCTGACTTCAACAGCG
oFH58 GAPDH qPCR reverse primer	IDT	ACCACCCTGTTGCTGTAGCCAA
oFH99 population dynamics reporter RT primer	IDT	GCTTGTAACTAATCTTGCGGCCG
oFH124 population dynamics reporter PCR primer with Illumina index N701	IDT	CAAGCAGAAGACGGCATACGAGATTCGCCTTAGTCTCGTGGGCTCGGAGATGTGTATAAGAGACAGTCCGTTACCTTGTTGCTGAGC
oFH125 population dynamics reporter PCR primer with Illumina index N702	IDT	CAAGCAGAAGACGGCATACGAGATCTAGTACGGTCTCGTGGGCTCGGAGATGTGTATAAGAGACAGTCCGTTACCTTGTTGCTGAGC
oFH135 population dynamics reporter PCR primer with Illumina index N703	IDT	CAAGCAGAAGACGGCATACGAGATTTCTGCCTGTCTCGTGGGCTCGGAGATGTGTATAAGAGACAGTCCGTTACCTTGTTGCTGAGC
oFH136 population dynamics reporter PCR primer with Illumina index N704	IDT	CAAGCAGAAGACGGCATACGAGATGCTCAGGAGTCTCGTGGGCTCGGAGATGTGTATAAGAGACAGTCCGTTACCTTGTTGCTGAGC
oFH137 population dynamics reporter PCR primer with Illumina index N705	IDT	CAAGCAGAAGACGGCATACGAGATAGGAGTCCGTCTCGTGGGCTCGGAGATGTGTATAAGAGACAGTCCGTTACCTTGTTGCTGAGC
oFH138 population dynamics reporter PCR primer with Illumina index N706	IDT	CAAGCAGAAGACGGCATACGAGATCATGCCTAGTCTCGTGGGCTCGGAGATGTGTATAAGAGACAGTCCGTTACCTTGTTGCTGAGC
oFH139 population dynamics reporter PCR primer with Illumina index N707	IDT	CAAGCAGAAGACGGCATACGAGATGTAGAGAGGTCTCGTGGGCTCGGAGATGTGTATAAGAGACAGTCCGTTACCTTGTTGCTGAGC
oFH140 population dynamics reporter PCR primer with Illumina index N708	IDT	CAAGCAGAAGACGGCATACGAGATCCTCTCTGGTCTCGTGGGCTCGGAGATGTGTATAAGAGACAGTCCGTTACCTTGTTGCTGAGC
oFH141 population dynamics reporter PCR primer with Illumina index N709	IDT	CAAGCAGAAGACGGCATACGAGATAGCGTAGCGTCTCGTGGGCTCGGAGATGTGTATAAGAGACAGTCCGTTACCTTGTTGCTGAGC
oFH142 population dynamics reporter PCR primer with Illumina index N710	IDT	CAAGCAGAAGACGGCATACGAGATCAGCCTCGGTCTCGTGGGCTCGGAGATGTGTATAAGAGACAGTCCGTTACCTTGTTGCTGAGC
oFH143 population dynamics reporter PCR primer with Illumina index N711	IDT	CAAGCAGAAGACGGCATACGAGATTGCCTCTTGTCTCGTGGGCTCGGAGATGTGTATAAGAGACAGTCCGTTACCTTGTTGCTGAGC
oFH144 population dynamics reporter PCR primer with Illumina index N712	IDT	CAAGCAGAAGACGGCATACGAGATTCCTCTACGTCTCGTGGGCTCGGAGATGTGTATAAGAGACAGTCCGTTACCTTGTTGCTGAGC
oFH126 population dynamics reporter PCR primer with Illumina index S501	IDT	AATGATACGGCGACCACCGAGATCTACACTAGATCGCTCGTCGGCAGCGTCAGATGTGTATAAGAGACAGGCTTGTAACTAATCTTGCGGCCG
oFH127 population dynamics reporter PCR primer with Illumina index S502	IDT	AATGATACGGCGACCACCGAGATCTACACCTCTCTATTCGTCGGCAGCGTCAGATGTGTATAAGAGACAGGCTTGTAACTAATCTTGCGGCCG
oFH145 population dynamics reporter PCR primer with Illumina index S503	IDT	AATGATACGGCGACCACCGAGATCTACACTATCCTCTTCGTCGGCAGCGTCAGATGTGTATAAGAGACAGGCTTGTAACTAATCTTGCGGCCG
oFH146 population dynamics reporter PCR primer with Illumina index S504	IDT	AATGATACGGCGACCACCGAGATCTACACAGAGTAGATCGTCGGCAGCGTCAGATGTGTATAAGAGACAGGCTTGTAACTAATCTTGCGGCCG
oFH147 population dynamics reporter PCR primer with Illumina index S505	IDT	AATGATACGGCGACCACCGAGATCTACACGTAAGGAGTCGTCGGCAGCGTCAGATGTGTATAAGAGACAGGCTTGTAACTAATCTTGCGGCCG
oFH148 population dynamics reporter PCR primer with Illumina index S506	IDT	AATGATACGGCGACCACCGAGATCTACACACTGCATATCGTCGGCAGCGTCAGATGTGTATAAGAGACAGGCTTGTAACTAATCTTGCGGCCG
oFH149 population dynamics reporter PCR primer with Illumina index S507	IDT	AATGATACGGCGACCACCGAGATCTACACAAGGAGTATCGTCGGCAGCGTCAGATGTGTATAAGAGACAGGCTTGTAACTAATCTTGCGGCCG
oFH150 population dynamics reporter PCR primer with Illumina index S508	IDT	AATGATACGGCGACCACCGAGATCTACACCTAAGCCTTCGTCGGCAGCGTCAGATGTGTATAAGAGACAGGCTTGTAACTAATCTTGCGGCCG
oFH151 population dynamics reporter PCR primer with Illumina index S510	IDT	AATGATACGGCGACCACCGAGATCTACACCGTCTAATTCGTCGGCAGCGTCAGATGTGTATAAGAGACAGGCTTGTAACTAATCTTGCGGCCG
oFH152 population dynamics reporter PCR primer with Illumina index S511	IDT	AATGATACGGCGACCACCGAGATCTACACTCTCTCCGTCGTCGGCAGCGTCAGATGTGTATAAGAGACAGGCTTGTAACTAATCTTGCGGCCG
oFH153 population dynamics reporter PCR primer with Illumina index S513	IDT	AATGATACGGCGACCACCGAGATCTACACTCGACTAGTCGTCGGCAGCGTCAGATGTGTATAAGAGACAGGCTTGTAACTAATCTTGCGGCCG
oFH154 population dynamics reporter PCR primer with Illumina index S515	IDT	AATGATACGGCGACCACCGAGATCTACACTTCTAGCTTCGTCGGCAGCGTCAGATGTGTATAAGAGACAGGCTTGTAACTAATCTTGCGGCCG
oFH180 viral barcode library extension primer	IDT	TGCATCGGTACCTCCGTTACCTTGTTGCTGAGCGGCGCGCC
oFH181 viral barcode library with viral index 1	IDT	CATGGAGCGGCCGCWSWSWSWSWSWSWSWSWSWSWSWSWSWGGATGGGCGCGCCGCTCAG
oFH182 viral barcode library with viral index 2	IDT	CATGGAGCGGCCGCWSWSWSWSWSWSWSWSWSWSWSWSWSWCTCATGGCGCGCCGCTCAG
**Recombinant DNA**		
PiggyBac Transposase Expression Vector	System Biosciences	PB210PA-1
pCDH-EF1α-MCS Lentivector	System Biosciences	CD502A-1
psPAX2	Addgene	#12260
pMD2.G	Addgene	#12259
Additional plasmids will be listed prior to publication	This study	N/A
**Software and algorithms**		
PyMol (v. 2.5.3)	Schrödinger	https://www.schrodinger.com/products/pymol
SerialEM (v. 3.9)	Mastronarde^79^	https://bio3d.colorado.edu/SerialEM/
DYNAMICS (v. XXX)	Wyatt Technologies	https://www.wyatt.com/products/software/dynamics.html
CFX Maestro (v. 4.0.2325.0418)	Biorad	https://www.bio-rad.com/en-us/product/cfx-maestro-software-for-cfx-real-time-pcr-instruments
STAR sequence read aligner (v. 2.7.8a)	Dobin et al.^80^	https://github.com/alexdobin/STAR
HTSeq-count (v. 0.13.5)	Anders et al.^81^	https://htseq.readthedocs.io/en/master/index.html
DESeq2 (v. 1.30.1)	Love et al.^83^	https://bioconductor.org/packages/release/bioc/html/DESeq2.html
R (v. 4.0.5)	R Core Team	https://www.r-project.org/
PANTHER (v. 17.0)	Thomas et al.^82^	http://www.pantherdb.org/
scikit-image (v. 0.19.2)	van der Walt et al.^84^	https://scikit-image.org/
Big-FISH (0.6.2)	Imbert et al.^85^	https://big-fish.readthedocs.io/
FlowJo (v. 10.8.1)	BD Biosciences	https://www.flowjo.com/
FLASH (v. 1.2.11)		https://ccb.jhu.edu/software/FLASH/
Starcode (v. 1.4)	Zorita et al.^87^	https://github.com/gui11aume/starcode
Custom analysis code	This study	https://github.com/felixhorns/RNA-export-2023

## Highlights

- RNA exporters package and secrete RNA within protective nanoparticles
- Engineered exporters achieve high specificity for cargo RNA
- Sequencing exported RNA barcodes enables monitoring of cell population dynamics
- Exporters enable cell-to-cell delivery and expression of mRNA cargo
