# Supplementary material for: Engineering RNA export for measurement and manipulation of living cells
Source: Cell. Author manuscript; Available in PMC 2023 Sep 27. (PMC10528933; doi:10.1016/j.cell.2023.06.013)

Figure S1. Design and characterization of engineered viral RNA exporters.

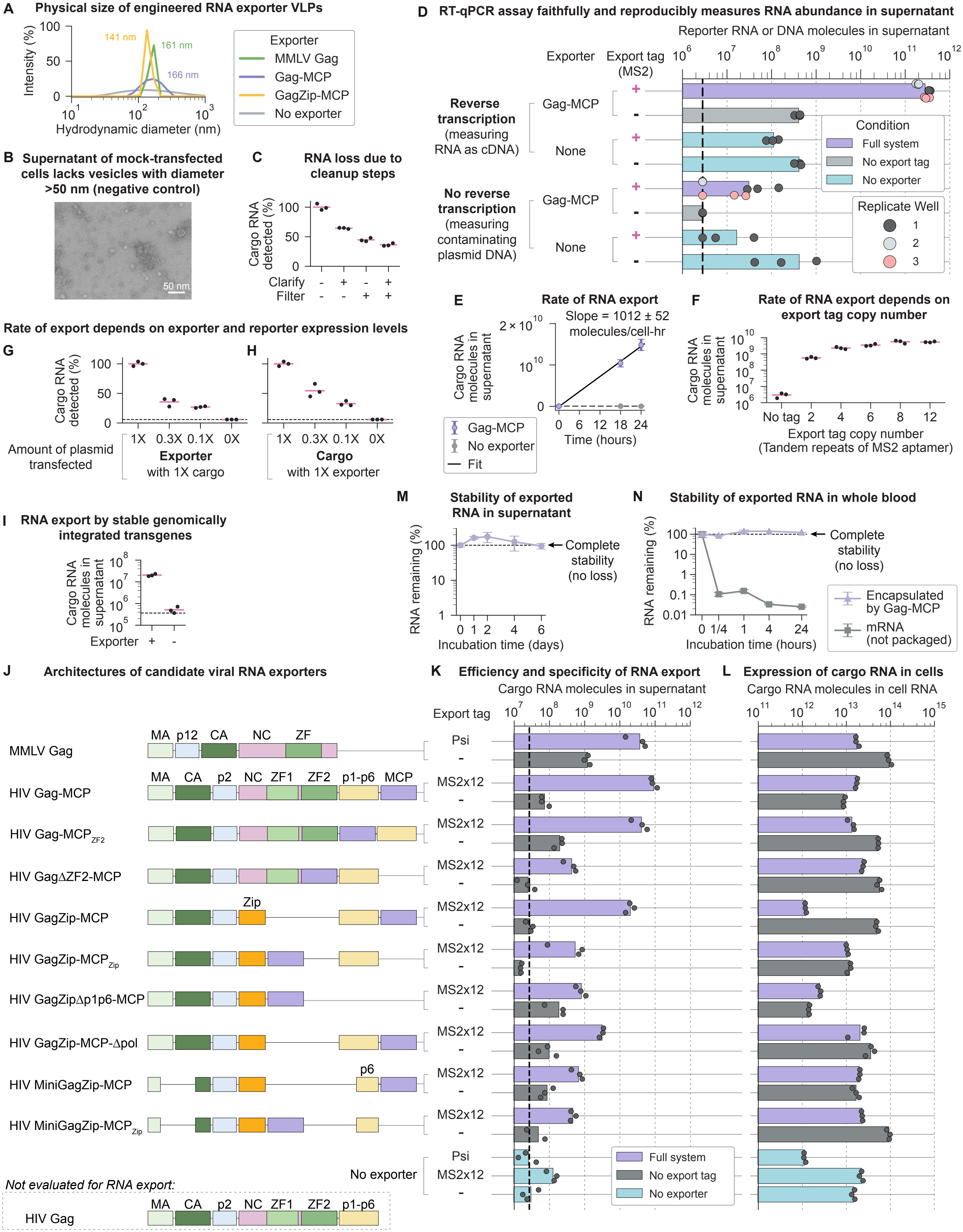

Figure S2. Design and characterization of protein nanocage-based RNA exporters.

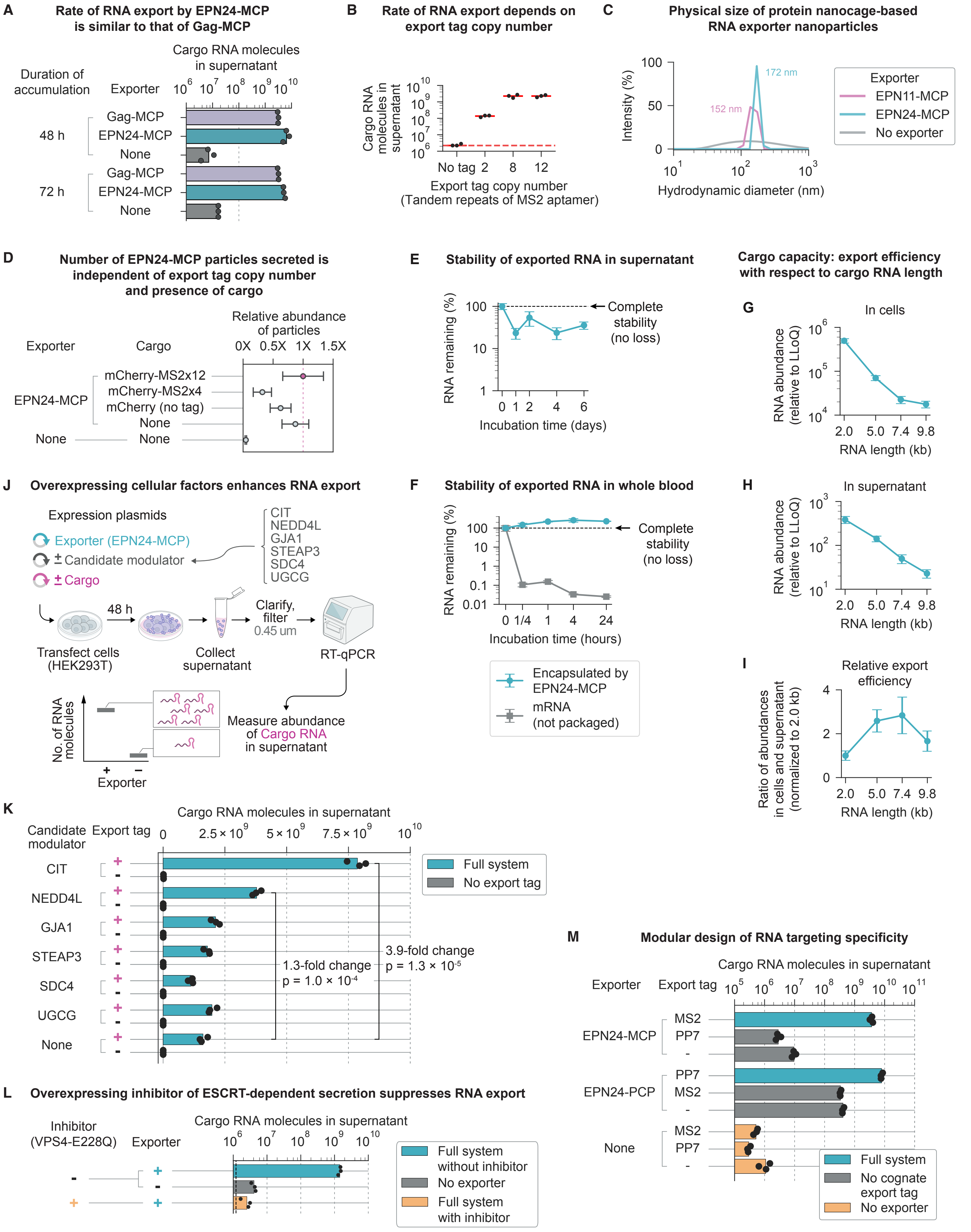

Figure S3. Additional genome-scale characterization of RNA export efficiency and specificity.

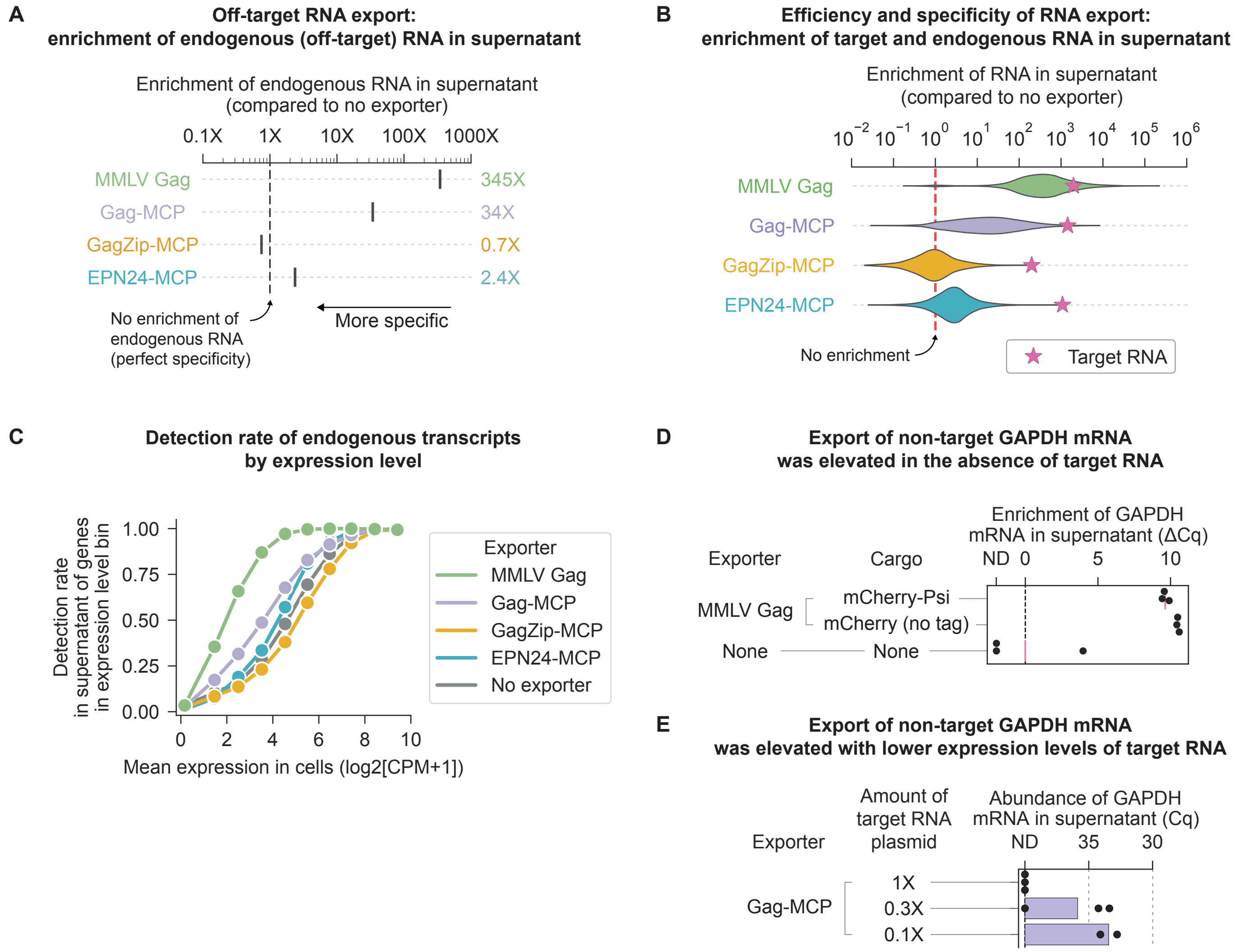

Figure S4. RNA exporters are non-toxic and do not perturb cellular morphology, growth, or transcriptome.

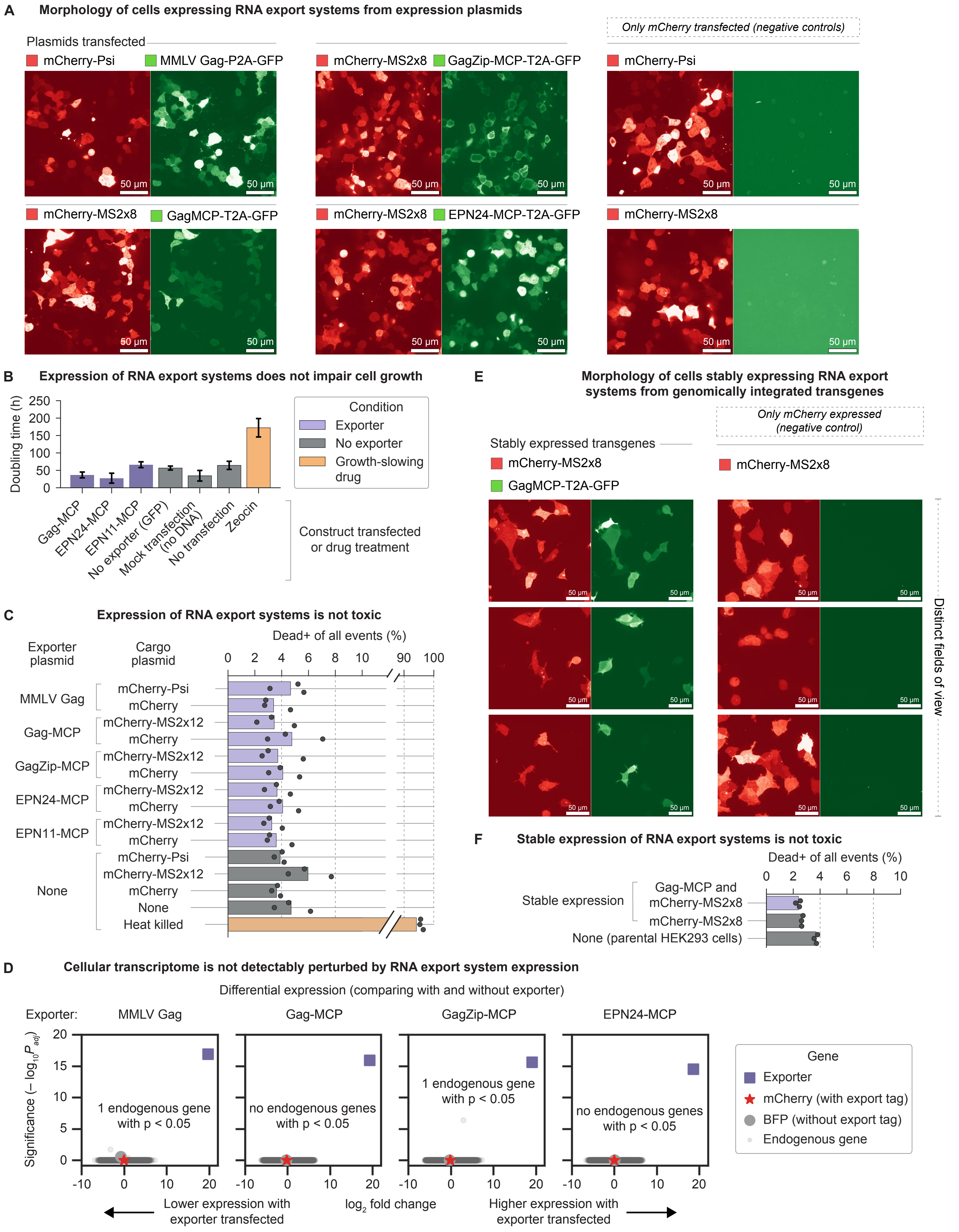

Figure S5. Barcode libraries, workflow, and performance of RNA-export based population dynamics reporter system.

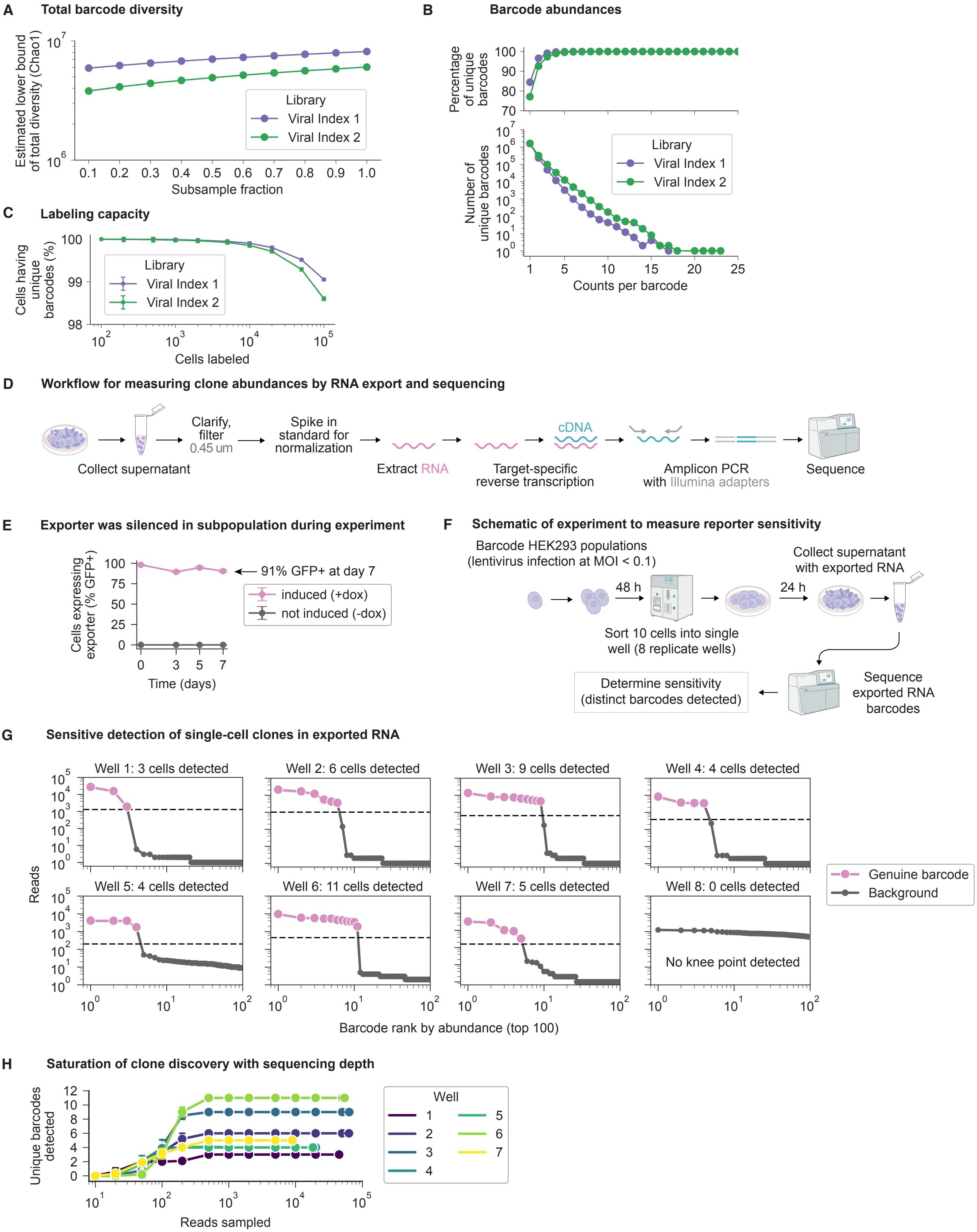

Figure S6. Additional characterization of monitoring population dynamics using RNA export and sequencing.

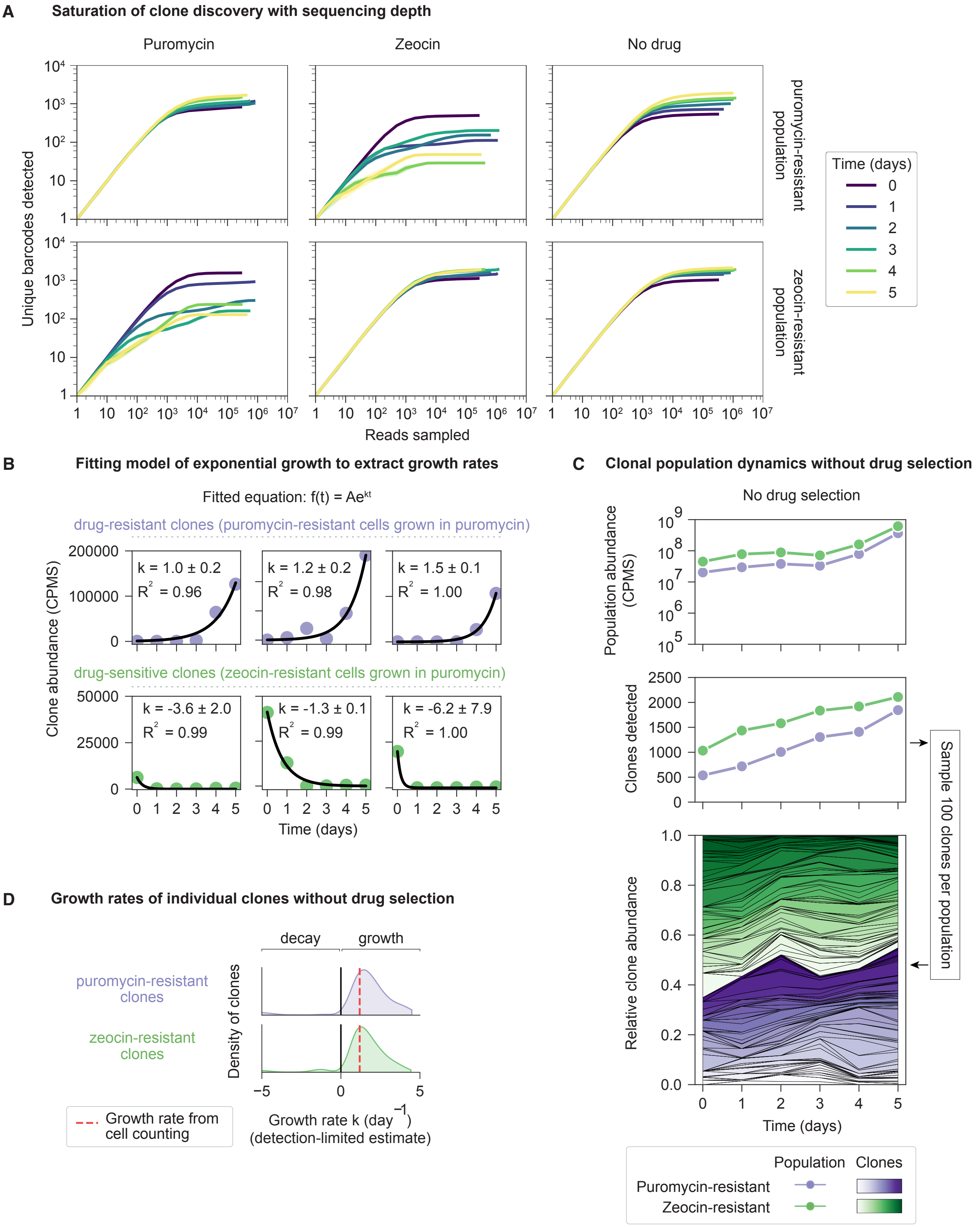

Figure S7. Optimizing and characterizing cell-to-cell delivery of RNA.

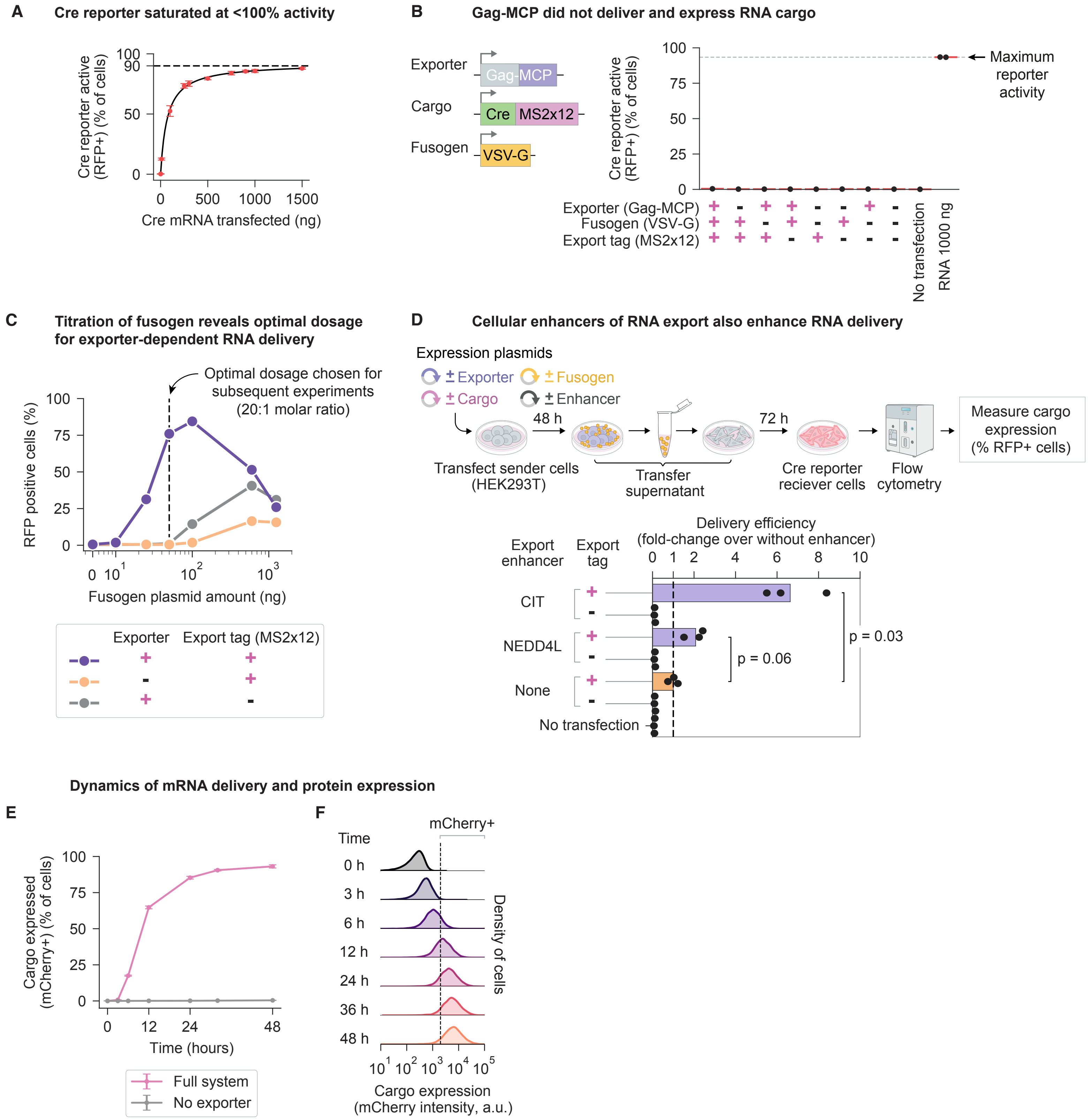

Supplement: 1 — Figure S1. Design and characterization of engineered viral RNA exporters, Related to Figure 1. (A) Diameter of virus-like particles (VLPs) secreted by cells expressing viral RNA exporters was measured using dynamic light scattering (DLS) after purification by ultracentrifugation through a 20% sucrose cushion. Labels indicate mean diameter. Note that particle diameters measured by DLS are larger than those measured by electron microscopy because aggregates cannot readily be distinguished from single particles by DLS. (B) Negative-stain transmission electron microscopy revealed that the supernatant of HEK293T cells transfected with expression plasmid of mCherry RNA alone (without an RNA exporter) lacked particles with >50 nm diameter. (C) Loss of RNA due to the cleanup steps of clarification (by centrifugation) and filtration was quantified using RT-qPCR. (D) To confirm that the RT-qPCR assay faithfully measures RNA abundance, rather than potential contaminants such as DNA plasmids, we omitted reverse transcription (RT) prior to qPCR. Omitting RT substantially reduced the apparent number of RNA molecules detected for all samples, indicating that the background signal from DNA contamination is lower than the foreground signal originating from RNA (cDNA after reverse transcription). Each dot represents one technical replicate; colors represent biological replicates (independent cell culture wells); bar indicates the mean of replicates. Consistency across biological replicates confirms the reproducibility of the assay. (E) Rate of RNA export was determined based on accumulation of RNA in culture supernatant after transfection using linear regression. Data for (E) are mean and standard deviation of three biological replicates. (F) Rate of RNA export can be tuned by varying the number of MS2 export tag repeats in 3’ UTR of the cargo RNA. (G) Rates of RNA export can also be tuned by the expression level of the exporter Gag-MCP and the cargo RNA, as shown by transfection of [file NIHMS1918572-supplement-1.pdf]
